# BRDT is an essential epigenetic regulator for proper chromatin organization, silencing of sex chromosomes and crossover formation in male meiosis

**DOI:** 10.1371/journal.pgen.1007209

**Published:** 2018-03-07

**Authors:** Marcia Manterola, Taylor M. Brown, Min Young Oh, Corey Garyn, Bryan J. Gonzalez, Debra J. Wolgemuth

**Affiliations:** 1 Department of Genetics & Development, Columbia University Medical Center, New York, NY, United States of America; 2 Human Genetics Program, Institute of Biomedical Sciences, Faculty of Medicine, University of Chile, Santiago, Chile; 3 Institute of Human Nutrition, Columbia University Medical Center, New York, NY,United States of America; 4 Department of Obstetrics & Gynecology, Columbia University Medical Center, New York, NY,United States of America; University of Pennsylvania, UNITED STATES

## Abstract

The double bromodomain and extra-terminal domain (BET) proteins are critical epigenetic readers that bind to acetylated histones in chromatin and regulate transcriptional activity and modulate changes in chromatin structure and organization. The testis-specific BET member, BRDT, is essential for the normal progression of spermatogenesis as mutations in the *Brdt* gene result in complete male sterility. Although BRDT is expressed in both spermatocytes and spermatids, loss of the first bromodomain of BRDT leads to severe defects in spermiogenesis without overtly compromising meiosis. In contrast, complete loss of BRDT blocks the progression of spermatocytes into the first meiotic division, resulting in a complete absence of post-meiotic cells. Although BRDT has been implicated in chromatin remodeling and mRNA processing during spermiogenesis, little is known about its role in meiotic processes. Here we report that BRDT is an essential regulator of chromatin organization and reprograming during prophase I of meiosis. Loss of BRDT function disrupts the epigenetic state of the meiotic sex chromosome inactivation in spermatocytes, affecting the synapsis and silencing of the X and Y chromosomes. We also found that BRDT controls the global chromatin organization and histone modifications of the chromatin attached to the synaptonemal complex. Furthermore, the homeostasis of crossover formation and localization during pachynema was altered, underlining a possible epigenetic mechanism by which crossovers are regulated and differentially established in mammalian male genomes. Our observations reveal novel findings about the function of BRDT in meiosis and provide insight into how epigenetic regulators modulate the progression of male mammalian meiosis and the formation of haploid gametes.

## Author summary

BRDT, a testis-specific member of the bromodomain and extra-terminal (BET) subfamily of epigenetic reader proteins, is essential for the generation of male gametes. In post-meiotic cells, BRDT is involved in chromatin organization and transcriptional regulation through its first bromodomain motif, as loss of the BD1 results in a truncated BRDT protein that fully interrupts the differentiation of the germ cells during the process of spermiogenesis. Complete loss of BRDT function results in an arrest during meiotic prophase with no cells progressing into post-meiotic stages. However, neither the specific role of BRDT in meiosis nor the pathways affected by its depletion are known. We investigated how BRDT controls meiosis by examining its subcellular localization during prophase I as well as the meiotic consequences observed with the loss of BRDT function. BRDT localizes throughout the chromatin of autosomes and sex chromosomes in a dynamic pattern during pachynema and diplonema. Loss of BRDT severely disrupts the epigenetic reprograming and silencing of transcription of the sex chromosomes, the global and regional chromatin configuration, and the formation and localization of crossovers in spermatocytes. Thus, BRDT regulates key meiotic processes that determine the genetic and epigenetic homeostasis of the male gamete.

## Introduction

The bromodomain is a highly conserved motif that recognizes and binds to acetylated lysine residues, a key step in reading epigenetic marks. Among the bromodomain-containing proteins, the BET (bromodomain and extra-terminal) subfamily is characterized by the presence of two bromodomains (hereafter referred to as BD1 and BD2) that bind to acetylated histones, and an extra terminal (ET) domain, which functions as a functional module for protein–protein interactions [1, 2]. In mammals, there are four BET members, BRD2, BRD3, BRD4 and BRDT, and among them, BRD4 and BRDT are structurally more similar in that they also have a region of homology at the carboxyl-terminus, referred to as the C-terminal domain (CTD). The BET proteins are epigenetic regulators with multiple functions in chromatin organization and transcriptional regulation. They direct the recruitment of diverse regulatory complexes to discrete regions in the genome, by mediating the tethering of protein complexes to acetylated histones and other proteins. In recent years, the BET proteins have received increasing attention due to their important implications in a wide range of human diseases [3], and thus, for being therapeutic targets of BET protein inhibitors [4].

BRDT is expressed uniquely in the testis, in late prophase I spermatocytes and spermatids [5]. BRDT has been shown in the mouse model to be essential for the normal progression of spermatogenesis: loss of BD1 results in a truncated BRDT protein and produces defects in spermiogenesis [6], with severely impaired chromatin organization in the spermatids [6, 7]. Moreover, complete loss of BRDT function (*Brdt-/-*) produces an arrest of spermatogenesis in meiosis and the absence of post meiotic cells [8]. Studies in humans have identified polymorphisms in the *BRDT* gene that are significantly associated with impaired spermatogenesis and male infertility [9, 10], suggesting that BRDT may contribute to idiopathic male infertility and could also be a potential druggable target for male contraception [11].

Biophysical experiments have shown that BD1 of BRDT binds to nucleosomes through a simultaneous recognition of acetylated histone tails and DNA, and that the nonspecific interaction of BD1 with the DNA facilitates the recruitment of BRDT to bulk chromatin [12]. In meiotic and post-meiotic cells, BRDT regulates gene expression, possibly by forming complexes with the P-TEFb components, cyclin T1 and Cdk9 [8]. In post-meiotic cells, BRDT has also been shown to function in post-transcriptional regulation [13], and allows the recruitment of chromatin remodeling complexes to promoters to regulate gene expression in a developmental-stage specific manner [14].

It has been increasingly proposed that epigenetic modifications play pivotal roles in modulating the progression of spermatocytes through the critical events of meiotic prophase I [15, 16]. Chromatin structure and epigenetic modifications have been shown to define recombination hotspots in the genome [17, 18], recruitment of recombinases [19], chromosomal interactions and segregation [20], and silencing of the sex chromosomes in spermatocytes [21–23]. However, which proteins mediate the recognition of the epigenetic modifications in meiosis and how these readers contribute to modulate the progression of prophase I are not well understood.

In the present study, we show that BRDT is an essential regulator of chromatin organization and reprograming during meiotic prophase. Loss of BRDT function disrupts the dynamics of chromatin modifications involved in maintaining the meiotic sex chromosome inactivation (MSCI), affecting the synapsis and silencing of the X and Y chromosomes. We also demonstrate that BRDT modulates the global chromatin organization of spermatocyte chromosomes and the local histone modifications of the chromatin close to the synaptonemal complex (SC). This function also influences the homeostasis of crossover (CO) localization and formation during pachynema, highlighting a possible epigenetic mechanism by which COs are regulated in mammalian genomes. Thus, BRDT may constitute a novel molecular pathway by which epigenetic regulators modulate the progression of male mammalian meiosis.

## Results

### During meiosis, BRDT is expressed from early pachytene to diplotene spermatocytes

**Fig 1:**
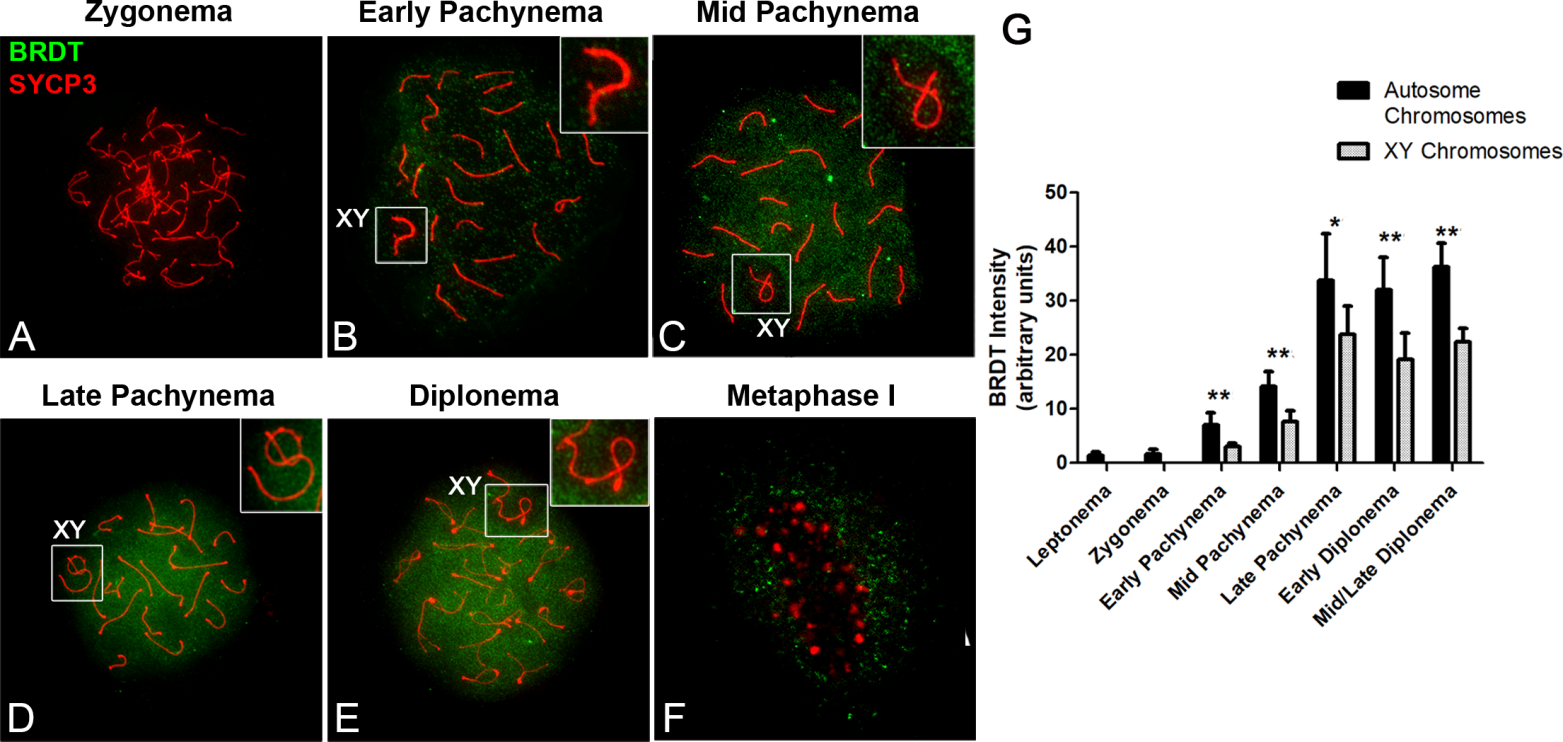
Pattern of BRDT protein expression during late stages of prophase I in male meiosis. (A-F) Immunolocalization of BRDT (green) and SYCP3 (red) during prophase I and metaphase I in WT spermatocyte spreads. Right upper insets show enlargements of the X and Y sex chromosomes (XY body) outlined in B—E. (A) BRDT signal is absent in zygonema. (B) BRDT is faintly detected in early pachynema. (C,D) In mid and late pachytene, and (E) diplotene spermatocytes, BRDT appears as a strong signal throughout the chromatin of autosome chromosomes and weakly in the XY body (upper right inset in C-E). (F) In metaphase I, BRDT is faintly observed in the cytoplasm. (G) Quantification of the signal intensity of BRDT in the chromatin of autosomes and sex chromosomes during each stage of prophase I. *p<0.05, **p<0.01. Error bars indicate standard deviation. Spreads were prepared from three 3 month-old WT mice; *n* = 10 leptotene, 10 zygotene, 20 early pachytene, 30 mid pachynema, late pachynema, early diplonema and mid/late diplonema spermatocytes respectively per each mouse.

Although we have previously reported that the *Brdt* gene is highly expressed in meiotic prophase spermatocytes, to gain a better understanding of the role of BRDT in meiosis in particular, we examined the developmental stage and sub-cellular localization of BRDT protein during meiotic prophase I in detail. To this end, we immunolocalized BRDT protein along with SYCP3, a marker of the axial elements (AE) of the synaptonemal complex (SC), which permitted clear identification of the meiotic chromosomes and classification of the stages of prophase I [24], in spermatocyte spreads from three 3 month-old wild type (WT) testes. BRDT protein was barely detectable, if at all, in leptotene and zygotene spermatocytes (Fig 1A and 1G), but started to be detected at early pachynema, mainly in the chromatin of autosomes (Fig 1B and 1G). By mid pachynema, BRDT was clearly detected throughout the chromatin of autosomes, but significantly less intensely in the sex chromosomes (Fig 1C, inset; 1G, **p<0.01). In late pachytene spermatocytes, BRDT protein was more robustly expressed as compared to earlier pachytene stages. That is, BRDT labeling appeared as a bright signal on the chromatin of autosomes and was slightly more intense in the X and Y chromosomes as compared to the sex chromosomes at early and mid pachynema (Fig 1D, inset; 1G, *p<0.05). This pattern persisted throughout diplonema, where BRDT was still strongly detected in the chromatin of autosomes and was significantly less intense in the chromatin of the X and Y chromosomes (Fig 1E and 1G, **p<0.01). At metaphase I, BRDT was barely observed in the chromatin, remaining primarily in the cytoplasm surrounding the chromosomes (Fig 1F). (Three 3 month-old WT mice, *n* = 10 leptotene, 10 zygotene, 20 early pachytene, and 30 mid pachynema, late pachynema, early diplonema and mid/late diplonema spermatocytes, respectively, per each mouse).

### Loss of BRDT causes apoptosis of late prophase I and metaphase I spermatocytes

In contrast to the defects in spermiogenesis seen in mice expressing a truncated protein lacking the BD1 of BRDT [6], complete loss of BRDT function (*Brdt-/-* mice) results in a total absence of spermatids in adult testes [8]. Indeed, *Brdt-/-* testes exhibited a significant decrease in the number of cells bearing H3 phosphorylated at serine 10 (H3S10Ph) [8], a mark associated with chromosome condensation during cell division [25], suggesting that a blockage might be occurring at the end of the meiotic prophase [8]. To more specifically elucidate which meiotic cells are lost in the absence of BRDT, we performed TUNEL staining in testicular sections from three 2 month-old WT and *Brdt-/-* mice, respectively. In WT adult testis, few apoptotic spermatocytes were observed and they were mainly in stage XII and I tubules, and were identified as late pachytene and metaphase spermatocytes, and early pachytene spermatocytes and step 1 round spermatids, respectively (S1 Fig). However, in *Brdt−/−* testes, TUNEL-positive spermatocytes were observed in all stages of tubules with pachytene and diplotene spermatocytes, with the most notably increased numbers in stages IX to XII (*p*<0.001; S1 Fig) and identified as mid pachytene to diplotene spermatocytes. The onset of apoptosis observed in these cells corresponded with the robust expression of BRDT normally observed in these meiotic stages. Interestingly, although it was previously reported that no post-meiotic spermatocytes were observed in the mutant testes [8], our very detailed analysis revealed occasional apoptotic pro-metaphase and metaphase spermatocytes in stage XII tubules in the *Brdt-/-* mice (S1 Fig). Clearly, however, the loss of BRDT largely affects the progression of late prophase I stages, confirming and extending previous observations [8].

### BRDT is dispensable for the synapsis and early DNA repair events of autosomes, but is essential for the synapsis of the sex chromosomes

**Fig 2:**
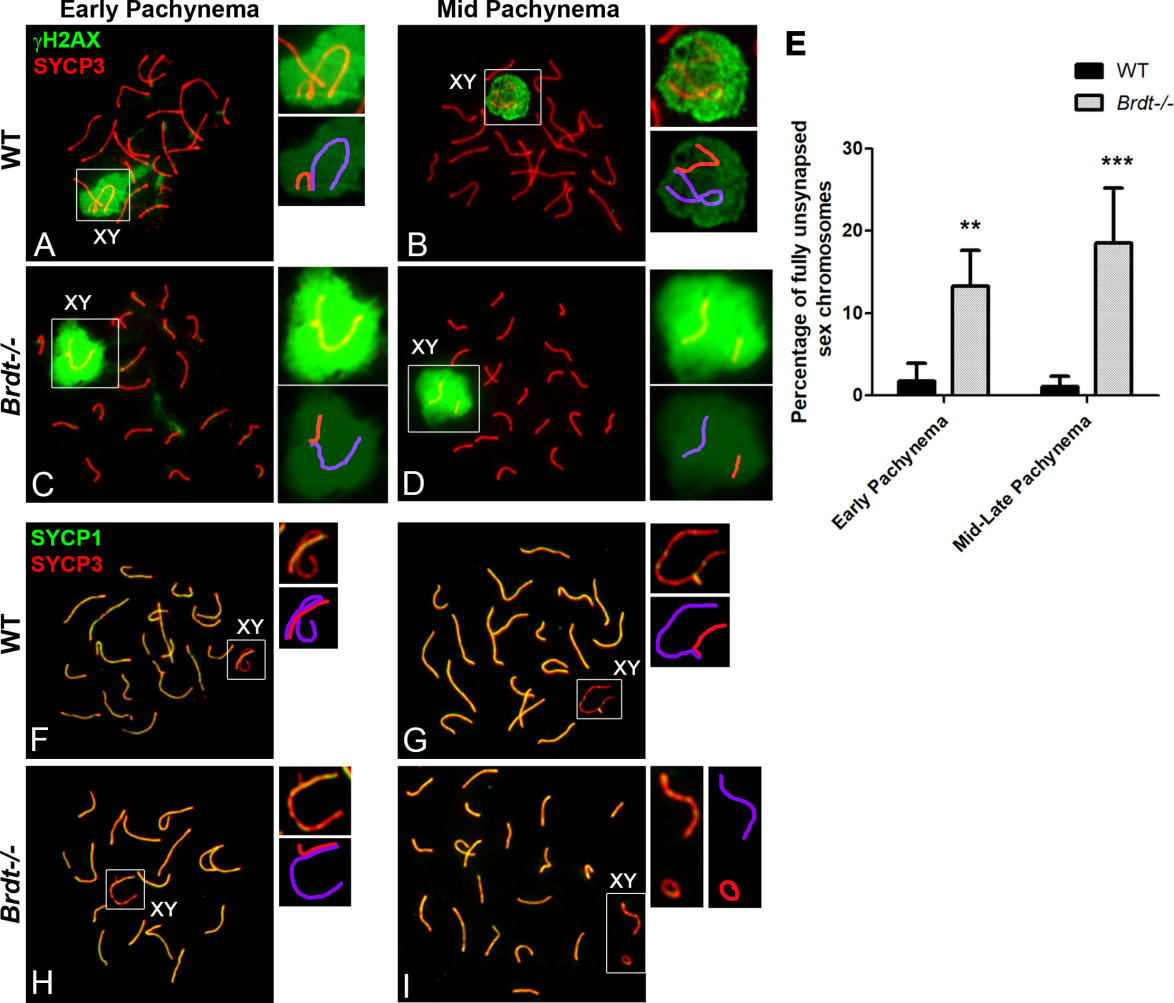
Depletion of BRDT does not affect the global DSB repair and synapsis of autosomes, but results in asynapsis of the sex chromosomes during pachynema. (A-D, F-I) Chromosome spreads of wild type (WT) and *Brdt-/-* early and mid pachytene spermatocytes. Upper insets show an enlargement of the sex chromosomes. A schematic representation of each inset is shown below the original, with the X chromosome represented in purple and the Y chromosome in red. (A-D) Immunolocalization of γH2AX (green) and SYCP3 (red). (A, C) In both WT and *Brdt-/-* early pachytene spermatocytes, γH2AX is present in the sex chromosomes and as foci in the chromatin adjacent to the SC. (B, D) At mid pachynema, γH2AX is observed only in the X and Y chromosomes, including those in complete asynapsis (D, inset). (E) Quantification of sex chromosomes with synapsis defects at the PAR in WT (black bar) and *Brdt-/-* (grey bar) spermatocytes. Samples were obtained from three WT and *Brdt-/-* 3 month-old mice. *n* = 62 and 99 early pachytene, and 125 and 132 mid/ late pachytene WT and *Brdt-/-* spermatocytes respectively. **p<0.01, ***p<0.001. (F-I) Immunolocalization of SYCP1 (green) and SYCP3 (red). In WT early (F) and mid pachynema (G), all chromosomes are fully synapsed, including the sex chromosomes. (H-I) *Brdt-/-* spermatocytes exhibit fully synapsed autosomal bivalents, but numerous sex chromosomes are completely unsynapsed (I, inset).

To begin to elucidate which defects are triggering the apoptosis of *Brdt-/-* spermatocytes, we first analyzed the efficiency of early DNA repair events by immunodetection of γH2AX as a marker of double strand breaks (DSBs), along with SYCP3, in spermatocyte spreads of *Brdt-/-* and WT mice. In both WT and *Brdt-/-* spermatocytes, γH2AX localized throughout all chromatin in leptonema and early zygonema (S2A and S2B Fig), indicating that normal DSBs are produced in the absence of BRDT. At early pachynema in both genotypes, γH2AX localization was restricted to a few foci in the chromatin close to the SC and extended over the chromatin of the sex chromosomes (Fig 2A and 2C). By mid pachynema (Fig 2B and 2D) to the end of diplonema, γH2AX persisted only in the X and Y chromosomes, indicating that the DSBs were repaired. However, despite the normal pattern of distribution of γH2AX, we regularly observed pachytene spermatocytes with synapsis defects in the sex chromosomes (Fig 2D).

To gain insight as to the extent and temporal nature of the synapsis defects of the X and Y chromosomes, we quantified the frequency of fully unsynapsed X-Y throughout the distinct phases of pachynema. We classified the sub-stages of pachynema using the appearance and distribution of γH2AX throughout the chromatin and the status of synapsis of the autosomes as criteria which have been previously described [24]. In early pachytene *Brdt-/-* spermatocytes, 86.7% of the pseudoautosomal region (PARs) of the X and Y were synapsed and concomitantly, 13.3 ±4.3% of the sex chromosomes were fully unsynapsed (Fig 2E, *p*<0.01) (*n* = 99 spermatocytes from three 3 month-old *Brdt-/-* mice). This percentage increased to 18.5 ±6.7% by mid to late pachynema (Fig 2E, *p*<0.001) (*n* = 132 spermatocytes from three 3 month-old *Brdt-/-* mice). The number of unsynapsed X-Y present in pachytene *Brdt-/-* spermatocytes was significantly different as compared to that in WT pachytene spermatocytes (Fig 2E, *p*<0.01, *p*<0.001 for early and mid/late pachynema, respectively), suggesting that synapsis of the sex chromosomes is significantly compromised by depletion of BRDT.

To evaluate if the regulation of DNA recombination by RAD51 was affected by depletion of BRDT, we next immunodetected RAD51 along with SYCP3, in WT and *Brdt-/-* spermatocytes and quantified the number of RAD51 foci in each stage of prophase I. In zygonema, both genotypes had similar numbers of mean RAD51 foci (162 ±22.5 and 166.3 ±14.6 foci in WT and *Brdt-/-*, respectively) (S3A and S3B Fig). In early pachynema, RAD51 foci were reduced in both WT and *Brdt-/-* (31.2 ±8.2 and 27.7 ±9.3 foci, respectively; *p =* 0.1) and by mid pachynema, the number of foci decreased to 15.6 ±6.9 and 17.6 ± 9.2 foci respectively (S3A and S3B Fig). By late pachynema and diplonema, RAD51 foci were rarely observed in WT and *Brdt-/-* spermatocytes (S3A and S3B Fig, *p* = 0.1) (*n* = 18 and 21 zygotene, 25 and 37 early pachytene, 100 and 200 mid and late pachytene WT and *Brdt-/-* spermatocytes, respectively, three 3 month-old mice per genotype).

Although no differences were observed in the total number of RAD51 foci between WT and *Brdt-/-* spermatocytes, because defects in DSB formation and RAD51 localization in the PAR are associated with synapsis defects in the X and Y [26, 27], we quantified the number of spermatocytes with a RAD51 focus or foci in the PAR in WT and *Brdt-/-* zygotene/early pachytene and mid pachytene cells. We observed that RAD51 focus/foci were properly formed in the PAR of *Brdt-/-* spermatocytes, with no significant differences as compared to WT spermatocytes (S3C Fig, *p* = 0.37)(*n* = 78 and 75 zygotene/early pachytene, and 85 and 89 mid pachytene WT and *Brdt-/-* spermatocytes, respectively; three 3 month-old mice per genotype). Thus, the analyses of γH2AX and RAD51 indicated that formation of DNA DSBs and homologous recombination *per se* were unaltered in the absence of BRDT. These results further suggest that repair of DNA DSBs was unaffected in *Brdt-/-* spermatocytes and is not likely involved in the synapsis defects exhibited by the sex chromosomes.

We next analyzed the dynamic pattern of chromosome synapsis throughout prophase I. Immunostaining *Brdt-/-* and WT spermatocyte spreads with SYCP3 and SYCP1, a marker of the central element (CE) of the SC, revealed that the timing of the progression of pairing and synapsis in leptonema and zygonema did not differ between WT and *Brdt-/-* spermatocytes (S2C and S2E Fig). At early pachynema, all homologous autosomes were completely synapsed throughout their entire length (Fig 2F and 2H). The lack of differences in the synapsis of autosomes between WT and *Brdt-/-* spermatocytes persisted until late pachynema (Fig 2G and 2I) and by the diplotene stage, desynapsis of the autosomes proceeds with no significant differences between WT and *Brdt-/-* spermatocytes (S2D and S2F Fig). In contrast to the normal synapsis exhibited by autosomes, the absence of BRDT gave rise to spermatocytes with synapsis defects of the X and Y chromosomes (Fig 2I), in sub-stages of pachynema where these chromosomes are normally fully synapsed through the PAR (Fig 2G). Taken together, these data show that the achievement and stabilization of the synapsis of the sex chromosomes, but not the autosomes, is affected in the absence of BRDT.

### Depletion of BRDT causes defects in the dynamics of repressive histone modifications in the sex chromosomes

In pachynema, the pairing and synapsis of the X-Y is thought to be maintained by formation of the MSCI [26]. Given the synapsis defects of the PAR observed in *Brdt-/-* spermatocytes, we next investigated whether the establishment of characteristic epigenetic modifications of MSCI might be affected in the absence of BRDT. As mentioned earlier, the temporal dynamics and localization of γH2AX is unaltered by depletion of BRDT, suggesting that initiation of MSCI may not be affected by BRDT (Fig 2C and 2D). However, BRDT may influence the second phase of epigenetic modifications that maintain the MSCI during pachynema and diplonema. To determine if the progression and maintenance of MSCI is BRDT-dependent, we assessed the dynamics of appearance of distinct and temporally regulated histone modifications characteristic of MSCI [28].

**Fig 3:**
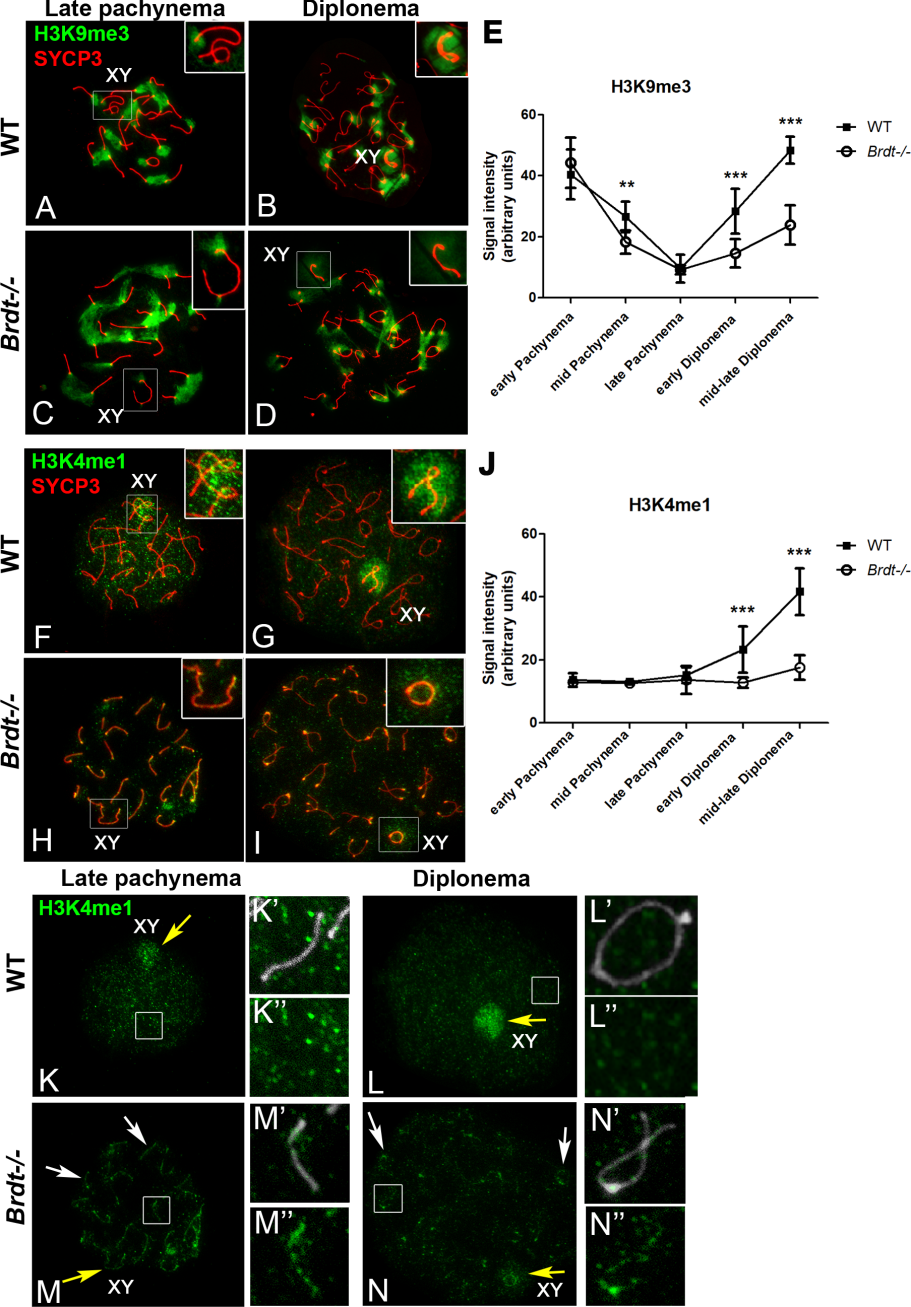
The dynamics of localization and incorporation of histone modifications linked to chromatin condensation/transcriptional repression is altered in absence of BRDT. (A-D, F-I) Chromosome spreads of wild type (WT) and *Brdt-/-* late pachytene and diplotene spermatocytes. Insets show an enlargement of the sex chromosomes. (A-D) Immunolocalization of H3K9me3 (green) and SYCP3 (red). (A, C) In late pachynema of both WT and *Brdt-/-* spermatocytes, H3K9me3 localizes only in the pericentric heterochromatin (PH) and as a very small signal in the PAR of the sex chromosomes. (B) In WT diplonema, H3K9me3 signal is intensely visualized in the PH and sex chromosomes. (D) In *Brdt-/-* diplotene spermatocytes, H3K9me3 staining persists in the PH but is weakly observed in the X and Y (insets). (E) Quantification of the signal intensity of H3K9me3 in the sex chromosomes in both WT (black square) and *Brdt-/-* (white circles) spermatocytes. Error bars indicate standard deviation. **p = 0.0072, ***p = 0.001. Samples were obtained from three 3 month-old WT and *Brdt-/-* mice. *n* = 10 and 17 early pachytene, 40 mid and 40 late pachytene, 30 early and 40 mid/late diplotene WT and *Brdt-/-* spermatocytes, respectively, per mouse. (F-I) Immunolocalization of H3K4me1 (green) and SYCP3 (red). (F) In WT late pachytene spermatocytes, H3K4me1 localizes throughout the chromatin of the autosomes and is slightly more intense in the sex chromosomes. (G) In diplonema, the signal intensity of H3K4me1 in the X and Y is notably increased (inset). (H) In *Brdt-/-* late pachytene spermatocytes, H3K4me1 signal starts to appear very faint throughout the chromatin from both autosomes and sex chromosomes (inset). (I) In diplonema, H3K4me1 remains weak in the X and Y (inset). (J) Quantification of the signal intensity of H3K4me1 in the sex chromosomes in both WT (black square) and *Brdt-/-* (white circles) spermatocytes. Error bars indicate standard deviation. *** *p* = 0.001. Samples were obtained from three 3 month-old WT and *Brdt-/-* mice. *n* = 13 early pachytene, 30 mid pachytene, 40 and 50 late pachytene, 30 early and 30 mid/late diplotene WT and *Brdt-/-* spermatocytes, respectively. (K-N) The same chromosome spreads of WT and *Brdt-/-* late pachytene and diplotene spermatocytes shown in F-I, but only H3K4me1 is displayed. (K’-N”) Enlargement of the chromosomes demarcated by the white rectangle in (K-N), with the chromosomes identified by SYCP3 (grey) in (K’-N’). (K-N) Yellow arrow indicates the sex body. (J-K”) In WT spermatocytes H3K4me1 is homogeneously distributed throughout the chromatin and is more concentrated in the sex chromosomes (yellow arrow). (M-N”) In the absence of BRDT, H3K4me1 is mainly located in the chromatin adjacent to the SC (white arrows, M-N”‘).

We first analyzed the temporal localization pattern of H3 trimethylated at lysine 9 (H3K9me3), a histone mark associated with chromatin condensation and transcriptional repression, and quantified the signal intensity in the sex chromosomes at different stages of prophase I. In mouse spermatocytes, H3K9me3 localizes at the pericentric heterochromatin throughout prophase I and exhibits a specific localization pattern in the sex chromosomes [28]. As expected, in WT spermatocytes, H3K9me3 localizes in both X and Y in early pachynema and is then restricted to the Y chromosome in the transition from early to mid pachynema. H3K9me3 progressively decreases until it disappears from the sex chromosomes during late pachynema (Fig 3A and 3E), but re-appears intensely in the unsynapsed chromatin of the sex chromosomes at diplonema (Fig 3B and 3E). However, in *Brdt-/-* spermatocytes the pattern of H3K9me3 is altered in later stages of prophase I. That is, at mid pachynema, H3K9me3 signal in the X-Y was significantly lower than in WT spermatocytes (Fig 3E, *p* = 0.0072). Although in *Brdt-/-* late pachytene spermatocytes H3K9me3 disappears from the sex chromosomes in a similar temporal pattern as WT cells (Fig 3C and 3E), the re-appearance of this histone mark in the X-Y at diplonema is notably weaker than that observed in WT diplotene spermatocytes (Fig 3D) and it never reached the normal levels that are observed in WT cells (Fig 3E, *p* = 0.001) (n = 10 and 17 early pachynema, 40 mid and 40 late pachynema, 30 early and 40 mid/late diplonema WT and *Brdt-/-* spermatocytes respectively per each mouse; three 3 month-old WT and *Brdt-/-* mice).

We next examined the dynamics of localization and signal intensity of histone H3 monomethylated at lysine 4 (H3K4me1), another modification related to transcriptional repression [29] and which has been shown to appear in the sex chromosomes by the end of the pachytene stage [28] (Fig 3F and 3G). Similar to H3K9me3, depletion of BRDT resulted in a defective localization of H3K4me1 in the sex chromosomes at the end of pachynema and diplonema (Fig 3H and 3I). Moreover, in *Brdt-/-* spermatocytes, the X and Y chromosomes exhibited significantly lower levels of H3K4me1 than those in WT spermatocytes during all late pachytene and diplotene stages (Fig 3J, *p* = 0.001) (n = 13 early pachynema, 30 mid pachynema, 40 and 50 late pachynema, 30 early and 30 mid/late diplonema WT and *Brdt-/-* spermatocytes respectively per each mouse; three 3 month-old mice per genotype). Analysis of H3K4me3 showed no observable differences between WT and *Brdt-/-* spermatocytes (S4A–S4H Fig). During the course of our analysis, we observed that in *Brdt-/-* spermatocytes, H3K4me1 localized closer to the SC of autosomes and sex chromosomes (Fig 3M–3N”), a pattern distinct from that seen in WT spermatocytes (Fig 3K–3L”). That is, from late pachynema (Fig 3M–3M”) through diplonema (Fig 3N–3N”), H3K4me1 was enriched in the chromatin loops closer to the SC in 76±11.8% of the spermatocytes analyzed (n = 100 WT and 110 *Brdt-/-* spermatocytes; three 3 month-old mice per genotype). Moreover, quantification of H3K4me1 signal intensity in the overall chromatin (not close to the SC) of *Brdt-/-* autosome chromosomes evidenced a significant reduction of 1.9-fold as compared to WT autosomes (*p* = 0.0021) (S5 Fig). This suggested that BRDT could be involved with determining the correct localization pattern of H3K4me1 in distinct chromatin regions of autosomes and sex chromosomes during late prophase I stages.

Although we do not exclude the possibility that the overall change in distribution of H3K4me1 in late prophase I *Brdt-/-* spermatocytes influences the reduction in its levels observed in the sex chromosomes, the higher fold-difference in H3K4me1 signal between WT and *Brdt-/-* sex chromosomes (5.1, *p* = 0.0006) (S5 Fig), compared to that in WT and *Brdt-/-* autosome chromosomes (1.9, *p* = 0.0021), suggested that failure of mechanisms specific to the MSCI might also be mediating the decrease of H3K4me1 in the X-Y in late prophase I *Brdt-/-* spermatocytes.

### BRDT mediates the removal of active transcriptional markers from the sex chromosomes during late prophase I stages

**Fig 4:**
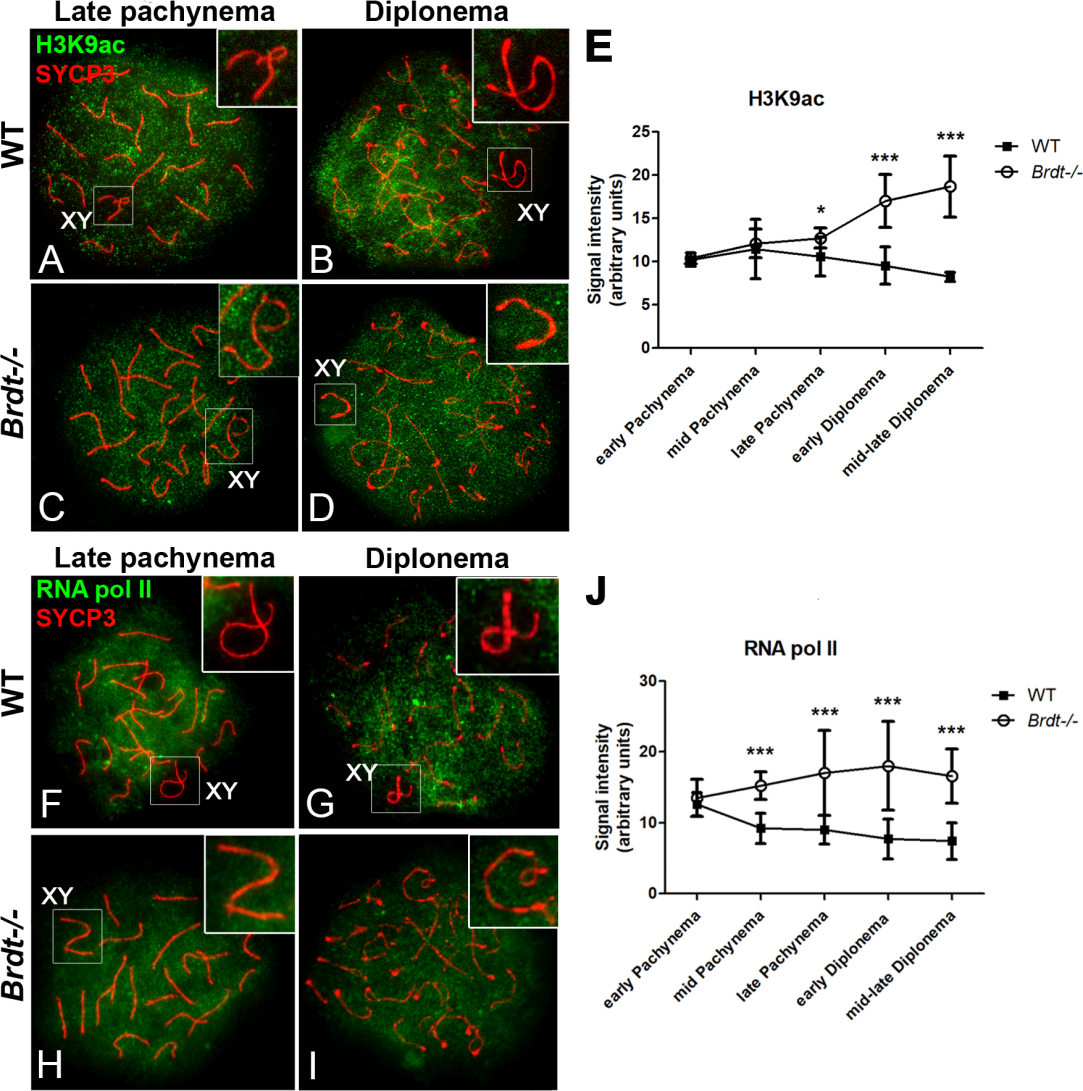
Depletion of BRDT disrupts the timing of removal of transcriptional activation markers in the X and Y chromosomes. (A-D, F-I) Chromosome spreads of wild type (WT) and *Brdt-/-* late pachytene and diplotene spermatocytes. Insets show an enlargement of the sex chromosomes. (A-D) Immunolocalization of H3K9ac (green) and SYCP3 (red). (A-B) In WT late pachytene and diplotene spermatocytes, H3K9ac localizes in the chromatin of autosomal chromosomes but is absent in the sex chromosomes (XY; inset). (C-D) In *Brdt-/-* late pachytene and diplotene spermatocytes, H3K9ac signal is intense and localizes in the chromatin of both autosome and sex chromosomes (XY; inset). (E) Quantification of the signal intensity of H3K9ac in the sex body in both WT (black squares) and *Brdt-/-* (white circles) spermatocytes. *p = 0.05, ***p = 0.001. Samples were obtained from three 3 month-old WT and *Brdt-/-* mice. *n* = 10 and 12 early pachytene, 24 and 25 mid pachytene, 30 late pachytene, 20 early and 40 mid/late diplotene WT and *Brdt-/-* spermatocytes, respectively, per mouse. Error bars indicate standard deviation. (F-I) Immunolocalization of RNA pol II (green) and SYCP3 (red). (F-G) In WT late pachytene and diplotene spermatocytes, RNA pol II localizes throughout the chromatin of autosomes but is barely detected in the sex chromosomes (XY, inset). (H, I) In *Brdt-/-* spermatocytes, RNA pol II is present in the chromatin of both autosomes and sex chromosomes. (J) Quantification of the signal intensity of RNA pol II in the sex chromosomes in both WT (black squares) and *Brdt-/-* (white circles) spermatocytes. ***p = 0.001. Samples were obtained from three 3 month-old WT and *Brdt-/-* mice. *n* = 15 early pachytene, 25 mid pachytene, 40 late pachynema, 25 early and 35 mid/late diplotene WT and *Brdt-/-* spermatocytes, respectively per mouse. Error bars indicate standard deviation.

The altered dynamics of histone modifications involved in the maintenance of MSCI during late prophase I stages raised the possibility that the temporal dynamics of localization of active transcriptional markers in the sex chromosomes is altered in *Brdt-/-* spermatocytes. To test this hypothesis, we first analyzed the localization of histone H3 acetylated at lysine 9 (H3K9ac), which associates with transcriptionally active chromatin. H3K9ac localizes in the chromatin of autosomes from mid pachynema to diplonema and is barely detected in the chromatin of the sex chromosomes during these stages [28] (Fig 4A and 4B). While the localization of H3K9ac in the autosomes is similar between WT and *Brdt-/-* pachytene and diplotene spermatocytes, its distribution in the X and Y chromosomes is altered (Fig 4C and 4D). That is, there was a consistent presence of H3K9ac in the sex chromosomes in mutant pachytene and diplotene spermatocytes, whereas it was not detected in the WT. Quantification of the signal intensity of H3K9ac in the sex chromosomes revealed that its levels in *Brdt-/-* spermatocytes actually increased throughout late prophase I stages, in contrast to the decrease that was observed in the X and Y in WT spermatocytes (Fig 4E, *p* = 0.05 and *p* = 0.001) (n = 10 and 12 early pachynema, 24 and 25 mid pachynema, 30 late pachynema, 20 early and 40 mid/late diplonema WT and *Brdt-/-* spermatocytes, respectively per mouse; three 3 month-old mice per genotype). This result suggested that the transcriptional repression of the sex chromosomes is affected by depletion of BRDT.

Indeed, immunolocalization of RNA pol II was barely detected in the sex body of WT spermatocytes [28] (Fig 4F and 4G), but showed an intense signal in the XY chromatin of *Brdt-/-* late pachytene and diplotene spermatocytes (Fig 4H and 4I). Quantification of the levels of RNA pol II protein signal in the sex chromosomes in *Brdt-/-* spermatocytes revealed that its levels were significantly higher than in WT XY chromatin (1.65 and 1.9 fold-change in mid and late pachynema, respectively, and 2.3 and 2.2 fold-change in early and mid/late diplonema, respectively; Fig 4J, *p* = 0.001) (n = 15 early pachynema, 25 mid pachynema, 40 late pachynema, 25 early and 35 mid/late diplonema WT and *Brdt-/-* spermatocytes, respectively per mouse; three 3 month-old mice per genotype). Collectively, these results showed that BRDT is involved in regulating the timing of appearance and disappearance of epigenetic modifications linked to the MSCI in the X and Y chromosomes during late prophase I stages.

### BRDT regulates the transcriptional silencing of the sex chromosomes

**Fig 5:**
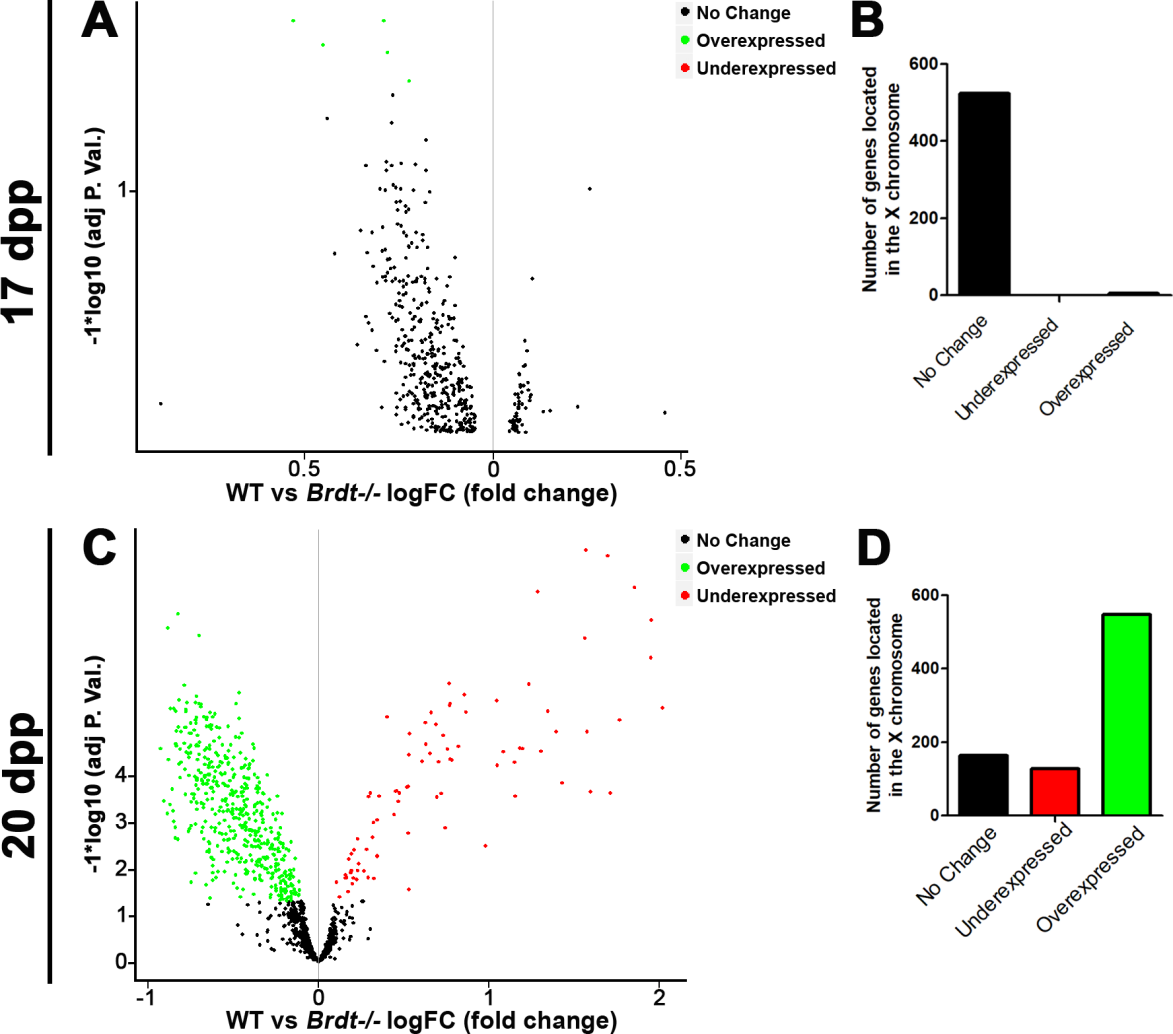
BRDT contributes to the transcriptional silencing of the X chromosome in spermatocytes. (A, B) Volcano plot representation of differentially expressed X-linked genes in *Brdt-/-* 17dpp (A) and 20dpp testes (B). The X axis represents the log fold-change (FC) in WT vs *Brdt-/-* spermatogenic cells. The Y axis represents the adjusted p-value. Only genes with an adjusted p-value of ≤0.05 were considered as differentially expressed genes (DEGs). (B, D) Number of X-linked genes with no change in expression, under-regulation or up-regulation in *Brdt-/-* 17dpp (B) and 20dpp (D) testes as compared to WT testes.

Our results indicate that BRDT influenced epigenetic modifications associated with silencing of the sex chromosomes, suggesting that BRDT could influence the transcriptional repression of sex-linked genes during the MSCI. To test this hypothesis, we analyzed the expression of all genes located in the X chromosome by mining an available transcriptomic database of *Brdt-/-* spermatogenic cells at 17 and 20 dpp (GEO number GSE39910) [8]. Importantly for our analyses, in these juvenile mice, the appearance of the phenotype of *Brdt-/-* mice is not yet visible, the number of spermatocytes is similar between WT and *Brdt*-/-, and the onset of apoptosis is not detected [8]. Among all the genes located in the X chromosome, we focused on genes that are transcribed only in testes, according to the mouse testes profile described by Schultz et al. [30]. Upon comparison with WT 17 and 20 dpp testes, the genes with a *p*-adjusted value below 0.005 and a fold-change of <1.5 were defined as differentially expressed genes (DEGs). Based on these criteria, in 17 dpp (Fig 5A and 5B; S3 Table), 525 genes showed no change in expression between WT and *Brdt-/-* testes, while only 5 were overexpressed in absence of BRDT. In 20 dpp testes (Fig 5C and 5D; S3 Table), from 838 genes analyzed we found 163 genes with no change in expression and 675 DEGs. Among them, 128 genes were under expressed and 547 genes were overexpressed. This suggests that in testes containing predominantly pachytene and diplotene spermatocytes, in which normally the sex chromosomes are subjected to chromosome-wide silencing, 65.3% of the genes located in the X chromosome were significantly overexpressed in absence of BRDT. This overexpression pattern appears to be characteristic of X-linked genes, contrasting with the overall downregulation pattern observed in all autosomes in *Brdt-/-* 20dpp testes (S6A Fig). We also note that most of the genes with no change in expression correspond to genes expressed in spermatogonia, which do not express BRDT and would not be expected to be affected by its loss.

To confirm the overexpression observed in the X-liked genes, we used RNA isolated from WT and *Brdt-/-* spermatocytes to quantify by qRT-PCR the expression of six genes located in different positions within the X chromosome, which in the transcriptomic analysis were overexpressed: *Ddx3x* (8.17cM), *Nxf2* (20.72cM), *Fmr1* (38cM), *Pet2* (39.95cM), *Gm5072* (40cM) and *Huwe1* (68cM), and *Scml2* (74cM). qRT-PCR showed that in *Brdt-/-* spermatocytes, the expression levels of *Nxf2*, *Fmr1*, *Gm5072*, and *Huwe1* were significantly higher than WT spermatocytes (S6B Fig, * *p*<0.05 and *** *p*<0.001). Interestingly, we noticed that genes located toward the distal end of the X chromosome, which contains the PAR, tended to be overexpressed. These gene expression studies show that BRDT influences the silencing of the sex chromosomes during meiosis.

### Depletion of BRDT changes the normal chromatin organization of spermatocytes and affects the length of the chromosome axes

**Fig 6:**
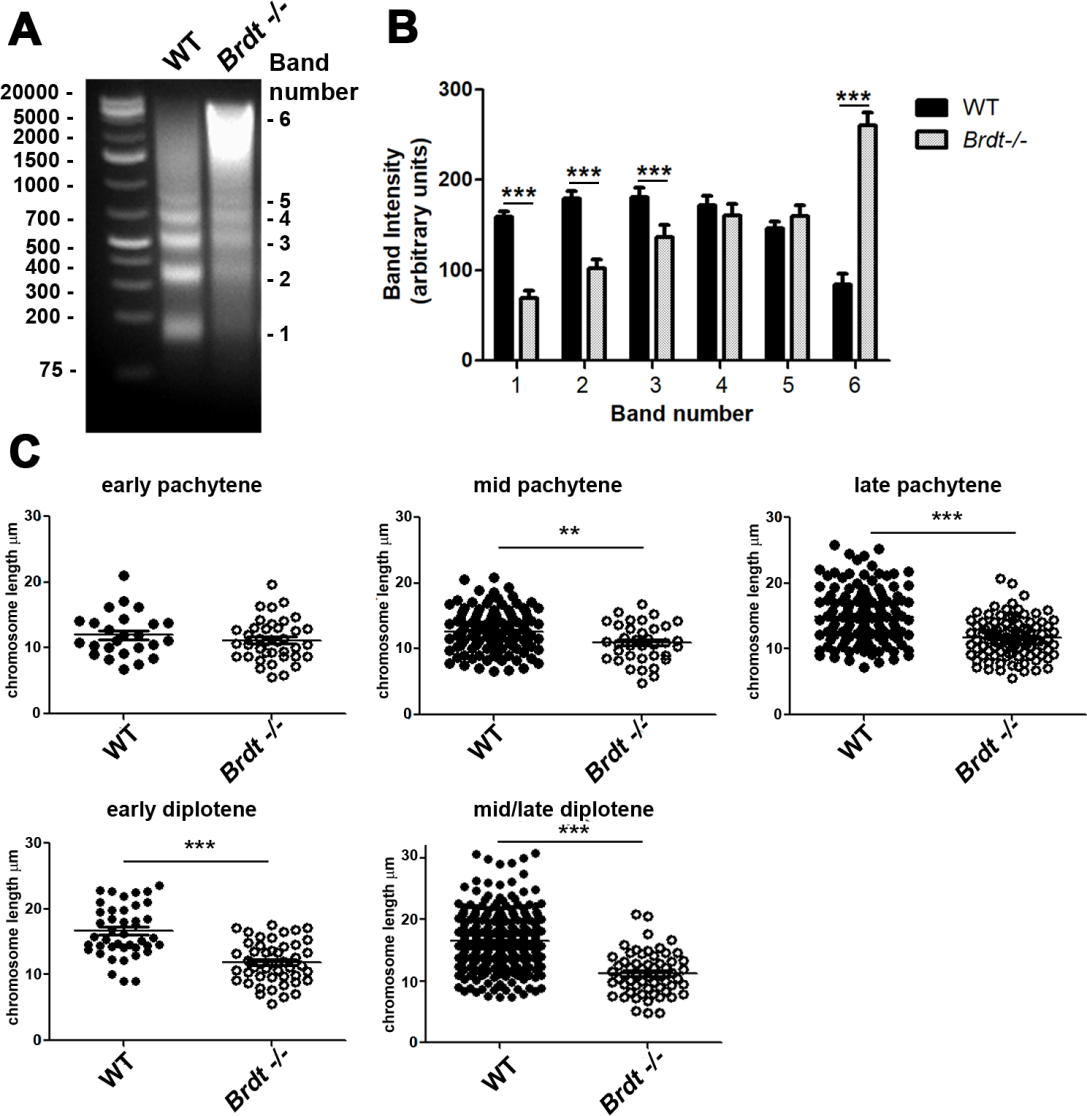
Absence of BRDT leads to a global chromatin condensation and a decrease in the length of the SC of autosomal chromosomes. Chromatin from seminiferous tubules from 17 day old WT and *Brdt-/- testes* was digested with 1U/ul MNase. In WT chromatin, MNase digestion produced low molecular weight bands corresponding mainly to mono- (band 1), di- (band 2) and tri- (band 3) nucleosomes. MNase digestion of *Brdt-/-* chromatin leads to digestion products with the bulk of chromatin ranging between 1500 and 4000 kb in size (bands 4 and 5) and a concomitant reduction in the mono-, di- and tri nucleosome fractions. Four WT and *Brdt-/-* mice were used per experiment; 3 experiments total. (B) Quantification of the intensity of the bands obtained after digestion with MNase of WT (black bars) and *Brdt-/-* (grey bars) chromatin. Error bars indicate standard deviation. *** *p*<0.001. (C) The length of each SC from autosome chromosomes in WT (black circles) and *Brdt-/-* (white circles) spermatocytes was measured and quantified in micrometers. **p<0.0028, ***p<0.0001. Error bars indicate standard deviation. Samples were obtained from three WT and *Brdt-/-* mice. *n* = 22 and 39 early pachytene; 92 and 45 mid pachytene, 147 and 105 late pachytene; 38 and 53 early diplotene, and 158 and 65 mid/late diplotene WT and *Brdt-/-* spermatocytes, respectively.

Given the altered pattern of H3K4me1 distribution observed during late prophase, we next asked if this reflected a change in the overall chromatin organization of the spermatocytes. To examine this possibility, we assessed chromatin accessibility in *Brdt-/-* and WT total testicular cells from 17 dpp by digestion with MNase. This age was selected because, as previously mentioned, testes at this age are highly enriched in pachytene spermatocytes and neither the number of meiotic cells nor the incidence of apoptosis present in the testis is significantly altered in the absence of BRDT [8]. We found that depletion of BRDT led to a more condensed chromatin structure in spermatocytes, with a large portion of the chromatin being inaccessible to MNase digestion (Fig 6A). That is, compared to WT cells, the digestion of *Brdt-/-* cells generated more high molecular weight DNA, which ranged between 1500 and 4000 kb in size (Fig 6A and 6B). There was a concomitant reduction of low molecular weight fragments, particularly the mono-, di- and tri-nucleosome fractions, indicating a decrease in the MNase cleavage between these nucleosome oligomers (Fig 6A and 6B, *p*<0.001; four WT and *Brdt-/-* mice each were used per experiment, 3 experiments per digestion).

A previous study reported that defects in chromatin organization can have pronounced effects in the length of the axis of a chromosome [31, 32]. Consideration of this model led us to propose that depletion of BRDT might also alter the length of the axes of meiotic prophase chromosomes. To test this hypothesis, we measured and quantified the chromosome axis length in early, mid, and late pachytene, and early and mid/late diplotene WT and *Brdt-/-* spermatocyte spreads. At early pachynema, the lengths of autosomal axes were similar in WT and *Brdt-/-* cells (average of 12.7 μm ±3.6 and 11.5 μm ±3.1 respectively (Fig 6C, *p =* 3.2; n = 22 WT and 39 *Brdt-/-* spermatocytes, three 3 month-old animals per genotype). However, by mid and late pachynema, there was a significant shortening in the axial lengths in BRDT-deficient as compared to WT autosome chromosomes (15 ±4.8% and 19.5 ±2.9% respectively, **p<0.0028, ***p<0.0001, respectively; n = 92 and 45 mid pachytene, and 147 and 105 late pachytene WT and *Brdt-/-* spermatocytes, respectively; three 3 month-old mice per genotype). Furthermore, at early and mid/late diplonema, this shortening was even more pronounced, with mean axial lengths that were 30.5 ±4.2% and 32 ±3.4% shorter than observed in WT spermatocytes, respectively (Fig 6C, ***p<0.0001; n = 38 and 53 early diplotene, and 158 and 65 mid/late diplotene WT and *Brdt-/-* spermatocytes, respectively; three 3 month-old animals per genotype). These results indicate that the chromatin organization mediated by BRDT influences the length of the chromosome axis in late prophase I stages. They also suggest that the meiotic events that are dependent on the context provided by the chromosome axis and chromatin loop size, such as CO formation [33], could be affected by depletion of BRDT.

### BRDT is essential for normal CO formation

**Fig 7:**
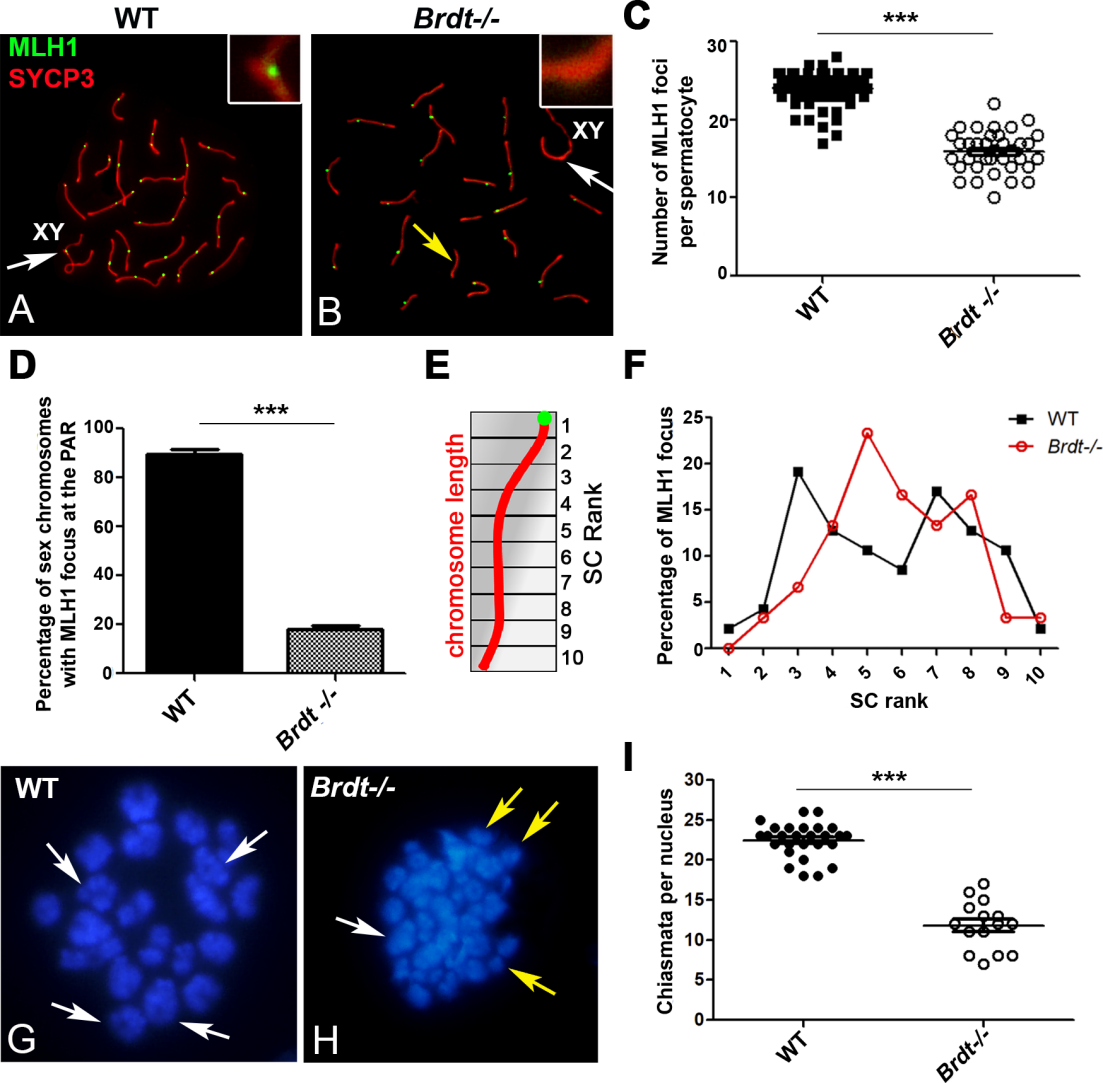
BRDT is required for the formation and distribution of CO during pachynema and chiasmata in metaphase. (A, B) Immunolocalization of MLH1 (green) and SYCP3 (red) in WT (A) and *Brdt-/-* (B) spermatocytes. Insets enlarge the PAR. White arrow indicates the presence or absence of the MLH1 focus in the PAR. Yellow arrow in (B) indicates autosomes with no MLH1 foci. (C) Quantification of the number of MLH1 foci per nucleus in WT (black squares) and *Brdt-/-* (white circles) pachytene spermatocytes. ***p<0.0001. Error bars represent standard deviation. Samples were obtained from three mice per genotype. *n* = 45 and 39 WT and *Brdt-/-* spermatocytes, respectively. (D) Quantification of sex chromosomes with an MLH1 focus in the PAR in WT (black bar) and *Brdt-/-* (grey bar) pachytene spermatocytes. ***p<0.0001. Error bars represent standard deviation. Samples were obtained from three each WT and *Brdt-/-* mice. *n* = 45 cells per genotype. (E) Scheme representing how each SC (in red) was divided into 10 equal length intervals (SC rank) from the centromere to the distal telomere (centromere is shown in green). (F) Relative distance of localization of each CO focus along autosomes in WT (black squares and line) and *Brdt-/-* (red circles and line) pachytene spermatocytes. Each point represents the percentage of MLH1 foci in each SC segment. (Samples were obtained from two mice per genotype. *n =* 15 WT and 15 *Brdt-/-* spermatocytes). Error bars represent standard deviation. (G,H) Chromosome spreads of diakinesis/metaphase I spermatocytes from WT and *Brdt-/-* mice stained with DAPI. White arrows show chromosomes with chiasmata. Yellow arrows in (H) show achiasmatic chromosomes. (I) Quantification of the number of chiasmata per nucleus in WT (black circles) and *Brdt-/-* (white circles) diakinesis/metaphase I spermatocytes (*n* = 6 and 4 respectively). ***p<0.0001. Error bars represent standard deviation. Samples were obtained from two mice per genotype. *n =* 4 and 6 WT and *Brdt-/-* spermatocytes, respectively.

Crossing over occurs and localizes in the context of both DNA and chromatin close to the SC. Given our observations of changes in the epigenetic organization of this chromatin as well as in the length of the chromosome axis in the absence of BRDT, we next determined whether CO formation during pachynema might also be altered, using immunolocalization of the late recombination marker MLH1 to foci. In WT spermatocytes, CO foci are found in all of the autosomes and in the PAR of the X and Y (Fig 7A), with an average of 24 ±2.5 CO foci per WT spermatocyte (Fig 7C; *n =* 45 spermatocytes, three 3 month-old WT mice). In contrast, depletion of BRDT resulted in a decrease in the number of MLH foci in the autosomes, with only 16.2 ±2.4 MLH1 foci per spermatocyte (Fig 7C, *p*<0.0001; *n* = 39 spermatocytes, 3 three month-old *Brdt-/-* mice). Indeed, some chromosomes completely lacked MLH1 foci, and there were very few chromosomes with two foci per chromosome (Fig 7B). Notably, the single MLH1 focus that is always produced in the PAR of the sex chromosomes decreased in frequency by 79.1 ±6.8% as compared to WT spermatocytes (Fig 7D, *p*<0.0001; *n* = 45 cells per genotype, three 3 month-old WT and *Brdt-/-* mice). This is of interest in light of the previously mentioned increase in defects of synapsis of the sex chromosomes observed in *Brdt-/-* spermatocytes, as CO formation in the PAR is believed to stabilize the synapsis of the X and Y [34].

We then examined if the COs formed in *Brdt-/-* spermatocytes are properly distributed within the autosomes and if CO interference, which ensures that COs are well-spaced along the chromosomes, was affected by the changes in chromatin organization and chromosome axis length produced by depletion of BRDT. To this end, we determined the distribution of MLH1 foci along the SCs from WT and *Brdt-/-* spermatocytes using analytical procedures described by Anderson et al. [35]. This method reveals the position of each MLH1-positive focus within the SC as its distance from the centromere (expressed as percent of the SC length), allowing the comparison of data from chromosomes of different lengths [35]. Thus, by dividing each SC in 10 equal length intervals (from centromere to distal telomere) (Fig 7E), it is possible to localize each MLH1 focus in distinct SC intervals. The distribution of MLH1-positive foci within the chromosomes is then obtained by taking the sum of the number of MLH1 foci detected in each interval. In WT spermatocytes, for SCs with one MLH1 focus, 72.1% of the COs were mostly distributed between intervals 3 and 9, with 19.1% ±3.4 and 17% ±2.6 of MLH1 foci concentrated in intervals 3 and 7 respectively, and 12.8% ± 3.1 and 12.8% ±2.9 localized in both intervals 4 and 8 respectively (Fig 7F; *n =* 15 cells, from two 3 month-old WT mice). There was a very low frequency of COs at the ends of the chromosomes, in agreement with the reported negative effect of pericentromeric heterochromatin as well as telomeres on CO formation [36, 37].

In *Brdt-/-* spermatocytes, however, there was a change in the position of the MLH1 foci along the SC, with the general distribution pattern shifted toward the distal end of the autosomes (Fig 7F) and the observation of most of the COs being distributed in fewer intervals as compared to WT. That is, 69.8% of the MLH1 foci were distributed between intervals 4 and 8; and the highest levels of foci were observed at intervals 5 (23.3% ±2.5), 6 and 8 (both with 16.6% ±1.9 and 16.6% ±2.7 respectively of the loci), rather than in intervals 3 and 7 in the controls (*n =* 15 cells, from two 3 month-old *Brdt-/-* mice). Similar to WT, MLH1 foci were not found in the intervals closest to the centromere and telomeres. Despite the change of localization of MLH1 foci in the SCs, it was possible to observe that COs were still well-spaced along the chromosomes in *Brdt-/-* spermatocytes. That is, when two foci were present on the same SC, both WT and *Brdt-/-* cells exhibited foci evenly distributed along the chromosomes, with an average separation between foci of 58.3 ±9.1 and 61 ±3, respectively. These observations suggest that BRDT may be involved in defining the formation and proper localization of COs in the chromosomes during pachynema, but it is dispensable for CO interference.

Consistent with these observations, analysis of the few mutant spermatocytes that reached diakinesis/metaphase I revealed a significant decrease in chiasmata in *Brdt-/-* compared to WT spermatocytes (12.7 ±2.1 versus 22.9 ±1.4 respectively) (Fig 7I, *p*<0.0001) (*n =* 4 and 6, from two WT and two *Brdt-/-* 3 month-old mice, respectively). Furthermore, while in WT cells all chromosomes formed chiasmata, all *Brdt-/-* diakinesis/metaphase I spermatocytes examined exhibited achiasmate univalent chromosomes (Fig 7G and 7H). Interestingly, qPCR analysis of genes that encode proteins that participate in establishing COs (*Mlh1*, *Msh4*, *Rnf212*, *Cntd1*, *Cdk2*, *Dmc1*, *Rad51*), were unaltered in *Brdt-/-* spermatocytes as compared to WT (S7 Fig). This result suggests that the defects in CO formation exhibited by depletion of BRDT are not likely to have been produced by defects in the transcription of genes involved in the DNA repair pathway of COs.

### BRDT facilitates a chromatin organization in the PAR hotspot that may be involved in proper CO formation

To determine if BRDT plays a role in promoting a regional chromatin organization in genomic regions containing CO sites, we took advantage of the unique characteristics of the PAR hotspot of the X chromosome and analyzed the chromatin organization of this region. The PAR hotspot was selected because it contains a well-defined cluster of overlapping hotspots that comprises 21.3 kb in length, at position 166,425 to 166,446 kb in the X chromosome [38]. The defined localization of the X-chromosome hotspots, together with the size of the PAR and the organization of the chromatin as short chromatin loops, generate a CO density that is at least 20 times higher than that found in autosomes [26]. This represents the ‘hottest’ cluster of DSBs in the mouse genome, and there is a CO formed in 99.9% of the cells [38]. This region was also selected because depletion of BRDT caused a significant reduction in the formation of the CO focus in the PAR (Fig 7D) and the histone modifications in the chromatin close to the SC of the X chromosome were altered (Fig 3M and 3N).

**Fig 8:**
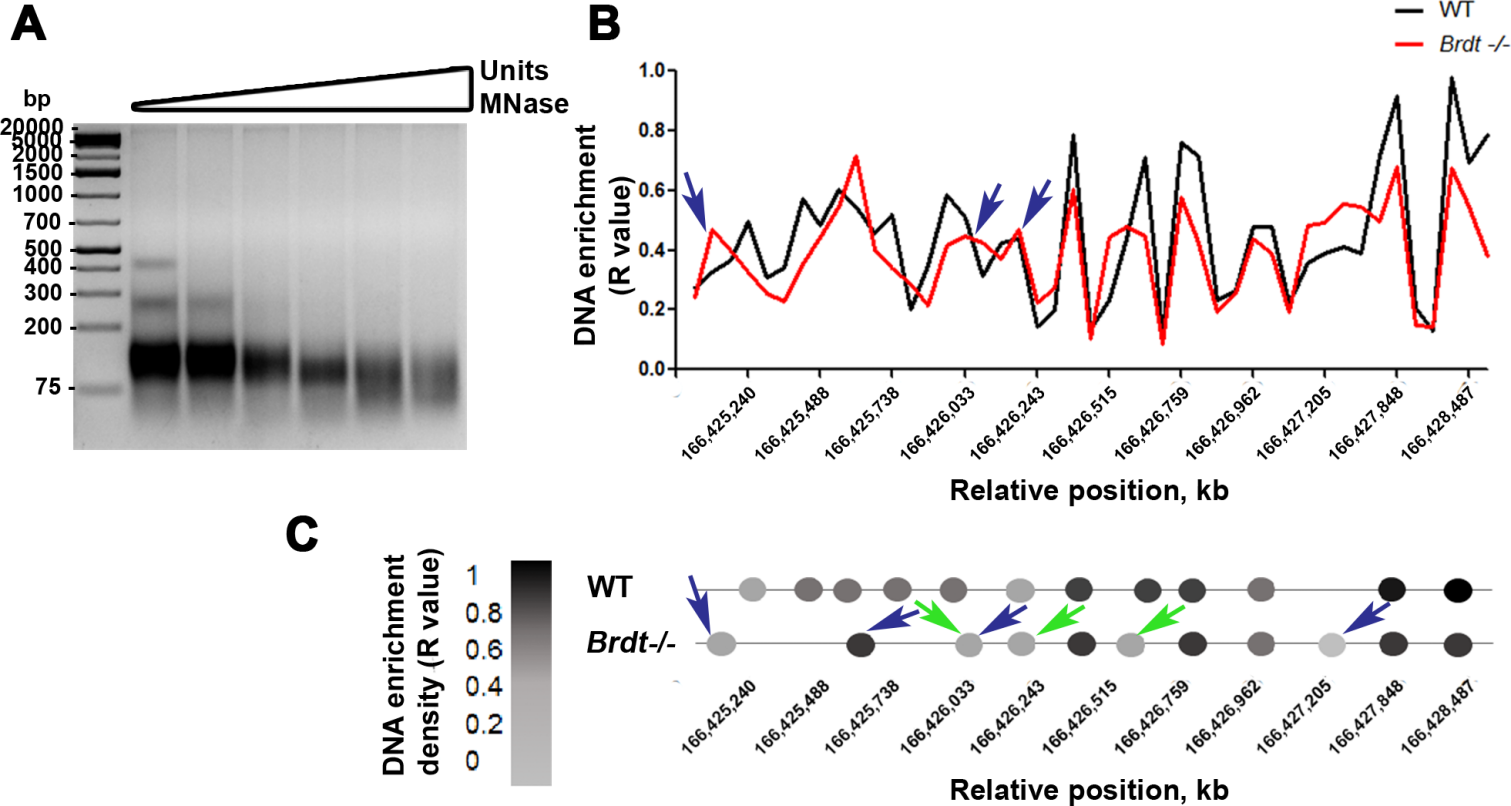
Chromatin organization at the CO hotspot of the PAR changes in the absence of BRDT. (A) Concentration-course of chromatin digestion from testicular cells with a 6 point MNase titration (0.125U/ul, 0.25U/ul, 0.5U/ul, 1U/ul, 2U/ul, 3U/ul). Samples were obtained from four WT mice per experiment. Three experiments total. (B) DNA enrichment density map of the region 166,425 to 166,446 kb of the X chromosome, obtained by tiled qPCR after MNase digestion of pachytene spermatocytes. Triplicates per each reaction were used in each genotype (four WT and *Brdt-/-* mice per experiment). (C) Graphic representation of the DNA enrichment position and density levels based on the graph displayed in (B) The color code represents the level of DNA enrichment based on the R value shown in (B). Blue arrows indicate protein occupancy areas present in *Brdt-/-* chromatin that are not observed in WT chromatin. Green arrows indicate lower protein occupancy areas in *Brdt-/-* chromatin.

We therefore isolated chromatin from enriched *Brdt-/-* and WT spermatocyte fractions and performed a DNA protein occupancy mapping analysis, based on MNase digestion of the chromatin, to obtain mononucleosome fractions (Fig 8A), followed by a nucleosome scanning assay (NuSA), which utilizes tiled qPCR [39]. Using this approach, we mapped a 3.3 kb region within the 21.3 kb PAR hotspot to define the occupancy of proteins on the DNA (Fig 8B).

We observed that in WT cells, protein occupancy is distributed all along the DNA and locates in mainly twelve DNA sites within the analyzed PAR hotspot region, separated by an average of 261 ±56.5 bp (Fig 8B and 8C). The higher peaks of protein occupancy (R value >0.75) were found between 166,426,342 to 166,428,487 bp, suggesting strongly positioned proteins in this region. Moderate peaks (R value 0.49–0.58) were found between 166,425,240 to 166,426,243 bp (Fig 8B and 8C). This chromatin organization pattern changed in the absence of BRDT. For example, localization, position and DNA enrichment levels (R value) were changed in the 21.3 kb PAR hotspot. From 166,424,993 to 166,426,243 bp, we found four areas of protein occupancy separated by an average of 510 ±249 bp, in contrast to the six areas observed in WT chromatin (Fig 8B and 8C). Moreover, in *Brdt-/-* chromatin, three of the four areas are not observed in WT chromatin (Fig 8C, blue arrows) and two of them corresponded to low protein occupancy areas (R value 0.46 and 0.42) (Fig 8C, green arrows). Also, from 166,426,515 to 166,428,487 bp, we observed a shift in the protection of some areas (166,426,515 bp and 166,427,205 bp) and a lower protection as compared to WT regions (Fig 8B and 8C). These data suggest that BRDT influences the normal chromatin architecture of the PAR hotspot.

## Discussion

The BET proteins are key epigenetic readers known to establish and modulate chromatin structure and organization, producing changes in the epigenomic landscape of cells [1, 2]. In the mouse, all four BET genes are expressed at unique stages of spermatogenesis [5], but their roles in this process are not well understood. The testis specific BET protein, BRDT, is expressed only in spermatocytes and spermatids, and its essential role in spermatogenesis has been clearly demonstrated by targeted mutagenesis in mouse models. Loss of the BD1 of BRDT impairs spermiogenesis [6] and complete loss of BRDT function blocks the progression of spermatocytes into the first meiotic division [8], both resulting in complete male sterility. Although the BD1 has been implicated in mediating chromatin remodeling and mRNA processing during spermiogenesis [7, 13] and BRDT has been implicated and changes in transcription during meiosis, the role of BRDT in specific meiotic functions is not well documented.

Our detailed analysis of BRDT expression during meiotic prophase revealed its restriction to late prophase I stages, mid pachynema to late diplonema, in particular. Interestingly, in contrast to the retention of other BET family members (BRD2 and BRD4) on mitotic metaphase chromosomes [40, 41], BRDT was not found to be significantly localized to meiotic metaphase I chromosomes. Indeed, the restricted expression pattern suggests that BRDT is dispensable for early meiotic events such as pairing and synapsis of autosomes, as well as for the global repair of DSBs. The restricted pattern also focused our attention on events that occur at these stages and impact the genetic and epigenetic stability of the sperm: namely, the formation and localization of COs, the behavior of the X and Y chromosomes, and the MSCI.

The significant percentage of sex chromosomes in complete asynapsis during pachynema that we observed suggests that the absence of BRDT impacts the synapsis process in the sex chromosomes, which could result from a failure to form stable synapsis or a premature desynapsis of the sex chromosomes prior to the end of pachynema. Our data further indicate that the asynapsis of the sex chromosomes is not a result of defective pairing of the X and Y, or lack of formation of DSBs in the PAR, as RAD51 foci are properly formed in the PAR of *Brdt-/-* spermatocytes. Alternatively, the defective synapsis of the PAR could be a consequence of defects in maintenance of the MSCI and the significant decrease of the CO formation in the PAR, both events that have been suggested to stabilize the synapsis of the X and Y in pachynema [26].

Efficient MSCI is important for normal progression of prophase I and failure of MSCI can lead to apoptosis via the pachytene checkpoint [42]. The spreading of γH2AX to the entire chromatin and the normal dynamics of RAD51 in the sex chromosomes suggest that BRDT is dispensable for the initiation of the MSCI. However, the defective establishment of repressive histone modifications in the X and Y indicate that BRDT is key for the epigenetic remodeling of the sex chromosomes, which is necessary for the maintenance of MSCI. It is thus possible that the failure of the sex chromosomes to achieve or maintain a proper synapsis and to maintain MSCI might trigger the robust apoptosis seen in late pachytene and diplotene spermatocytes in the absence of BRDT. Spermatocytes that do reach stable synapsis of the PAR are then able to progress until the end of diplotene, but are characterized by defective epigenetic changes in maintenance of MSCI of the X and Y as observed with H3K9me3, H3K4me1 and H3K9ac.

Whether BRDT has a direct or indirect role in maintaining the MSCI remains to be determined. Indeed, as BRDT is also involved in transcriptional activation during meiosis [8], we do not discard the possibility that BRDT could play an indirect role in the MSCI through transcription of other factors involved in epigenetic reprograming of the chromatin. Recent studies have shown that X-linked genes expressed in germ cells play key roles in regulating male fertility and that alteration in their expression or mutations in the genes may be associated with infertility in men [43]. We showed that BRDT regulates the silencing of the X-linked genes and its depletion produces an overexpression of 65.3% of these genes. In fact, our data showed that *Nxf2* and *Tex11*, two genes which have been shown to be essential for the regulation of meiosis and spermatogenesis, are overexpressed in *Brdt-/-* spermatocytes. NXF2-deficient mice exhibit defects in spermatogonia proliferation as well as defective spermatid formation, resulting in male infertility [44]. TEX11 promotes chromosome synapsis and interestingly, regulation of CO formation [45]. This is of particular interest as the phenotypes of *Tex11-/-* and *Brdt-/-* spermatocytes overlap in some features, such as asynapsis of the PAR, decrease in CO number, and defective chiasmata formation. This raises the possibility of a molecular link between TEX11 and BRDT and/or that the overexpression of TEX11 could potentiate the phenotype of *Brdt-/-* spermatocytes.

Our results further suggest that BRDT is also essential to maintain proper higher order chromatin structure during meiosis. That is, we observed that BRDT promotes proper histone modifications in the chromatin close to the SC and an overall open chromatin configuration in spermatocytes. The overall chromatin condensation observed in *Brdt-/-* spermatocytes is indeed consistent with the overall downregulation exhibited by autosomal genes that we observed and is in agreement with the previous report which showed a massive downregulation of genes in 20dpp testes in the absence of BRDT [8]. In addition, it has been shown that the structural maintenance of chromosomes influences the size of the chromatin loops located close to the SC [32, 46] and that variation in the length of the SC changes the number and length of DNA loops that are anchored to the SC [26, 31]. This is relevant because it is in the context of the chromatin loops that DSBs, recombination, and COs occur. Indeed, longer SC lengths have been correlated with higher numbers of COs per chromosome [35]. We thus speculate that the reduction in the length of the chromosomal SCs observed during mid and late pachynema contributes to the reduced number and altered distribution of COs within the chromosomes in *Brdt-/-* spermatocytes.

COs are also influenced by the local and higher order chromatin organization of the chromosomes [47]. In this context, the modulation of BRDT in local and global chromatin configuration and epigenetic marks might also be contributing to the successful formation of the COs in mammalian male meiosis. The defects in CO formation observed in *Brdt-/-* spermatocytes could be involved in triggering the apoptosis of spermatocytes at late pachynema. For those spermatocytes that achieve late diplonema to the metaphase 1 transition, defective COs producing achiasmatic chromosomes which could activate the metaphase checkpoint and trigger another wave of apoptosis of *Brdt-/-* spermatocytes [48].

As mentioned earlier, our analyses revealed that early recombination pathway events appear normal, and the fact that no chromosome fragmentation was observed suggests that DSBs are properly repaired in *Brdt-/-* spermatocytes. However, the reduction in number and abnormal localization of MLH1 foci indicate that BRDT is likely involved with CO formation in meiosis. One possibility is that BRDT modulates the maturation of the designated COs, by ensuring that the machinery that either promotes or antagonizes COs is properly recruited to the CO sites within the chromosomes [49]. Transcriptional analysis of genes encoding CO-related proteins revealed no changes in their expression, suggesting that BRDT is probably not involved in the regulation of expression of key factors that promote maturation and establishment of the COs. Whether BRDT has a direct role in recruiting CO complex proteins or indirect role by providing an epigenetic and chromatin structure suitable for the recruitment of the CO machinery proteins remains to be determined.

An additional observation about the function of BRDT in crossing over is that it appears to affect the proper localization/distribution of MLH1 foci within autosomes. The few MLH1 foci that are formed in absence of BRDT are distributed in regions that are different than those in WT chromosomes. However, CO interference is not affected in the chromosomes as our results also show that the distance between two crossover foci, when formed in *Brdt-/-* spermatocytes, is conserved independent of the change in SC length. This finding raises an interesting issue, as it has been shown that CO interference is part of a pattern shaped by the SC [31]. CO interference is not confined uniquely to the CO, but is related to SC nucleation and structure, which impacts the threshold-based designation and spreading interference process of the final COs [50]. Indeed, the structure of the SC provides a platform that promotes not only CO formation but also its inhibition, which limits the number of CO [51].

BRDT has no detectable role in SC formation *per se*; hence, the spreading pattern that COs might have within the SC, which determines CO interference, is unchanged by depletion of BRDT. Even changes in SC length at later prophase I stages are still not enough to modify this pattern. However, and despite the compromised position of CO sites in the chromosomes SC, the final formation and localization of a CO is produced by other mechanisms in which the chromatin and epigenetic landscape might play a determinant role. We propose that BRDT is a key element in regulating these mechanisms. These data indeed support previous results showing that CO formation is independent of CO interference [52]. Our findings concerning the role of BRDT in controlling CO formation and distribution in mammalian male meiosis provides a link between a male-specific epigenetic factor and the establishment of COs in specific regions of the chromosomes. Interestingly, they further suggest a possible mechanism by which epigenetic factors differentially expressed in males and females are potential mediators of the sexual dimorphism in recombination and CO patterns that occurs in mammalian meiosis [53].

The finding that lack of BRDT produces multiple alterations in different processes during prophase I, as well as the reported misregulation of gene expression such as of *Ccna1*, a key regulator of the diplotene to metaphase I progression [8], suggests there may be a combinatory set of defects which causes a progressive cell death in response to different checkpoints during prophase I and metaphase I. In conclusion, we have observed important functions for BRDT in regulating chromatin configuration and reprograming essential for MSCI and formation and localization of CO in spermatocytes, key meiotic processes that determine the genetic and epigenetic homeostasis of the gamete and the variability among individuals. Our observation of failure of these meiotic functions of BRDT provides insight into the infertility in the *Brdt* mouse models [6, 8] and in individuals reported as carrying polymorphisms in the *BRDT* gene [9, 10], as well as the possibility that BET protein inhibitors might be considered as a non-hormonal approach to male contraception [11].

## Materials and methods

### Mouse strains

All experiments involving mice were approved by the Columbia University Institutional Animal Care and Use Committee and performed in accordance with the National Institutes of Health guidelines for the care and use of animals. B6JTyr;B6N-Brdt^tm1a(EUCOMM)Wtsi^/Wtsi *(*hereafter named *Brdt-/-)* mice were obtained from the International KnockOut Mouse Consortium, Welcome Trust Sanger Institute, UK. Construction of the vector and characteristics of the mutation were previously described by Skarnes et al. [54] and Gaucher et al. [8]. B6JTyr;B6N mice were used as wild type (WT) strain.

### Tissue preparation and TUNEL staining of apoptotic cells

Testes from three 2 month-old WT and *Brdt-/-* mice were fixed in 10% formalin overnight at 4°C and then washed in 70% ethanol. Fixed and paraffin-embedded tissues were sectioned at 4 μm and serial sections were obtained. One section was used for staging the mouse seminiferous epithelium, by staining with hematoxylin along with immunostaining for γH2AX to identify and classify spermatocytes according to the different stages of prophase I. The serial section was then counterstained with hematoxylin along with TUNEL staining, performed using an *in situ* cell death detection kit (Roche Diagnostics) as previously described [55]. Only clearly stained cells were considered as apoptotic and only tubules cut perpendicular to the length of the tubule (round tubules in sections) were evaluated. 200 tubules per testis were analyzed per each genotype and the number of spermatocytes in each stage of the seminiferous tubule and the TUNEL-positive cells per cross-sectioned tubule were counted. Results were expressed as the percentage of TUNEL-positive spermatocytes/ total spermatocytes per each stage of the seminiferous tubules. Significant differences were assessed by statistical analysis using non-parametric Mann Whitney test with the threshold of significance set at 0.05.

### Preparation and immunofluorescence of spermatocyte spreads

Spermatocyte spreads were prepared following the procedures described by Manterola et al. [24]. Briefly, seminiferous tubules were isolated, placed in a petri dish, and mechanically disaggregated using forceps. Then, 80 to 200 μl of 100 mM sucrose was slowly added and mixed with the cells. From this suspension, 14 μl was dropped onto a slide previously submerged in 1% paraformaldehyde (PFA), pH 9.2, and spread throughout the slide. The slides were then slowly dried in a humid chamber for 3h, washed with Photo-Flo 0.08% in distilled water, dried, and stored at −80°C until their use. The slides were then placed in PBS and incubated with the following primary antibodies: mouse anti-SYCP3 1:200 (Abcam, Ab12452); rabbit anti-SYCP3 1:200 (Abcam, Ab15093); rabbit anti-BRDT 1:100 [6]; rabbit anti-trimethyl histone H3 (Lys 9) 1:300 (EMD Millipore 07–442); rabbit anti-histone H3 (acetyl K9) 1:200 (Abcam, Ab4441); rabbit anti-monomethyl histone H3 (Lys4) 1:200 (EMD Millipore ABE1353); rabbit anti-trimethyl histone H3 (Lys4), clone CMA304 1:50 (EMD Millipore 05–1339); mouse anti-RNA pol II CTD4H8 1:200 (Upstate 05–623); mouse anti-γH2AX (Ser139) 1:1000 clone JBW301 (Upstate 05–636) (EMD Millipore); mouse anti-MLH1 1:100 Clone G168-15 (BD pharmingen, 550838); rabbit anti-RAD51 1:200 (Calbiochem, PC130) and human anti-centromere 1:300 (Antibodies Incorporated, 15-235-0001). After rinsing in PBST (phosphate-buffered saline, 1% Tween-20), the slides were incubated with the appropriate secondary antibodies diluted 1:200 in PBS as follows: Alexa 488-conjugated donkey anti-rabbit IgG (H+L), Alexa 488-conjugated donkey anti-mouse IgG (H+L), Alexa 594-conjugated donkey anti-mouse IgG (H+L), Alexa 594-conjugated donkey anti-rabbit IgG (H+L), Alexa 647-conjugated donkey anti-human IgG (H+L). Slides were counter-stained with DAPI and mounted with Prolong (Thermofisher). Chromosome spreads were examined using a Nikon eclipse E800 microscope equipped with epifluorescence optics, and the images were photographed with a high-definition cooled color camera head DS-Fi1c. All images were processed with Adobe Photoshop CS5 software.

### Criteria of prophase I staging and quantification of BRDT, H3K4me1, H3K9me3, H3K9ac and RNA pol II signal intensities in spermatocyte spread preparations

The different meiotic stages were identified by following previously described criteria [24] [28]. Briefly, we identified changes in chromosome morphology of autosomes and sex chromosomes, such as the appearance of excrescences on the AEs of sex chromosomes and the widening of SC attachment plates on the autosomes, as well as the synapsis behavior of autosomes and sex chromosomes, with special regard to the length of the pairing region between X and Y chromosomes.

To measure the signal intensity of BRDT, we analyzed a total of 480 spermatocytes from three 3 month-old wild type mice, distributed in 10 leptonema, 10 zygonema, 20 early pachynema, 30 mid pachynema, late pachynema, early diplonema and mid/late diplonema spermatocytes respectively per each mouse. For measurement of the signal intensity of each histone modification H3K9me3, H3K9ac, H3K4me1 as well as for RNA polymerase II (pol II) in the sex chromosomes, we analyzed an average of 450 spermatocytes from three 3 month-old wild type and *Brdt-/-* mice, From these, an average of 20 corresponded to early pachynema, 30 to mid pachynema, 40 to late pachynema, 30 to early diplonema and 30 to mid/late diplonema spermatocytes per each protein, per mouse and per each genotype.

BRDT, H3K9me3, H3K9ac, H3K4me1 and RNA polymerase II (pol II) intensities were quantified by selecting 5 areas of the immunofluorescent signal in the XY body and measuring the intensity of the signal using the measurement/mean value tool in ImageJ (NIH). For BRDT and H3K4me1 signals, 5 random areas in the chromatin of autosome chromosomes were also quantified. Both immunofluorescent signals and background were quantified and the final intensities were calculated by subtracting the background immunofluorescent signal from the immunofluorescent signal from the protein. Results represent the mean±SD. It is important to note that in spread preparations, apoptotic cells exhibit a particular morphology that does not resemble a normal spermatocyte [56] and thus, we discarded those cells from our analysis.

Statistical significance between mice was calculated by the one-way analysis of variance (ANOVA), followed by Tuckey post test. A *Z-*test for two proportions was calculated to determine statistical differences between the signal intensity of BRDT in the chromatin of autosomes and in the chromatin of the sex chromosomes. The threshold of significance was set at 0.05 with a confidence interval of 95%. Statistically significant differences between wild type and *Brdt-/-* spermatocytes were determined using a non-parametric Mann Whitney test with the threshold of significance set at 0.05. No statistically significant differences in the distribution of the results between animals of the same genotype were observed.

### Measurement of autosomal chromosome lengths and the relative distance of localization of each CO focus along the chromosomes

The lengths of autosomes of WT and *Brdt-/-* early, mid, and late pachytene, and early and mid/late diplotene spermatocyte spreads were measured by using the measurement tool in ImageJ (NIH), calibrated to micrometer (μm) by setting a known distance according to the microscope and digital camera. The position of the MLH1 foci along the autosome chromosomes of WT and *Brdt-/-* mid and late pachytene spermatocyte spreads was measured by determining the relative distance of each MLH1 focus in each SC using distance (percentage of SC length) from the centromere and distal telomere, according to the method and criteria described by Anderson et al. [35]. Ten mid and ten late pachytene WT and *Brdt-/-* spermatocyte spreads (*n* = 3 animals per each genotype), immunolabeled with SYCP3, MLH1 and centromere, were selected for measurement based on i) the presence of unbroken and unstretched SC, and ii) the presence of at least 19 MLH1 foci in the case of WT cells, and 10 MLH1 in *Brdt-/-* spermatocytes. Each SC was measured using the measurement tool in ImageJ (NIH), and the length was divided into 10 equal (10%) intervals. Using the same measurement tool, the position of each MLH1 foci was calculated by its distance to the centromere and to the distal telomere, respectively. The relative interference distance between two MLH1 foci on an SC was determined by following the method described by Anderson et al. [35].

### Isolation of enriched populations of spermatocytes and gene expression analysis

Preparation of enriched populations WT and *Brdt-/-* pachytene spermatocytes was performed according to our laboratory's established protocols [14] by using seven WT and sixteen *Brdt-/-* 2–3 month-old mice. The purity of cell populations was by flow cytometric analysis on a Becton Dickinson FACScan Flow Cytometer. Pools of primary spermatocytes were obtained at a purity of >85%. RNA was isolated by using a Qiagen RNeasy Micro Kit and one microgram of total RNA was used to prepare cDNA with random hexamer primers and the SuperScriptIII First-Strand Synthesis System (Invitrogen, #18080–051). Quantitative real-time PCR (qPCR) was performed according to our standard protocol [57]. Primer sets are provided in S1 Table. The *acidic ribosomal phosphoprotein P0* (*Arbp*) gene was used as an internal control for data normalization and the fold change between wild type and mutant samples calculated. All samples were analyzed in triplicate. Statistical differences of the expression of each gene between WT and *Brdt-/-* pachytene spermatocytes was performed using 2-way Anova with Bonferroni posttests.

### Transcriptomic analysis of the X chromosome

The transcriptomic analyses of the X chromosome were performed by using gene expression data from *Brdt-/-* testes performed by Gaucher et al [8] and obtained using the Illumina mouse WG-6 V2.0 gene expression array. The raw data from 17dpp and 20dpp WT and *Brdt-/-* mice was obtained from Gene Expression Omnibus, access number GSE39910, platform GPL6887. Within this page, we used the tool “Analyze with GEO2R” to establish the differential expression of genes by using the R package “Limma”, which uses a linear model to evaluate the differential expression and an empirical bayes method to moderate the standard errors of the estimated log-fold changes. The genes with an adjusted p-value of ≤0.05 and an absolute fold-change of <1.5 were considered as differentially expressed genes (DEGs). All the dataset generated, including DEGs and genes with no differential expression (no-change) were then grouped per chromosome by using the genome database biomaRt, obtaining the chromosome localization and expression pattern (DEG or no-change) for each gene. From the obtained list, we used R to filter the genes that were only expressed in testes by using the transcript profile of developing mouse testes described by Scultz et al. [30]. Ggplot2 was used to generate the graphs of the gene-expression profile of the X chromosome at 17dpp and 20dpp testes.

### Micrococcal Nuclease (MNase) sensitivity assay and quantification of digested DNA bands

Nuclei were isolated from testes from twelve 17 day-old WT and *Brdt-/-* mice, which are enriched for pachytene and diplotene spermatocytes. As these testes are in the first spermatogenic wave, they contain no spermatids (in the case of WT animals), and very few apoptotic cells (in the case of *Brdt-/-*) [8]. The MNase sensitivity assay and salt extraction analysis of chromatin were performed by following the procedure described by Kishi et al. [58], with few modifications. Briefly, seminiferous tubules from 6 testes (per each genotype) were suspended in 5 ml of buffer A (0.32 M sucrose, 15 mM HEPES-NaOH (pH 7.9), 60 mM KCl, 2 mM EDTA, 0.5 mM EGTA, 0.5% bovine serum albumin (wt/vol), 0.5 mM spermidine, 0.15 mM spermine and 0.5 mM dithiothreitol) and ground using a Dounce tissue grinder. The cells were then filtered through a 40 μM mesh filter and the resulting suspension was layered on a cushion of 45 ml buffer A containing 30% of sucrose (wt/vol) and centrifuged at 3,000 g for 10 minutes at 4°C. Then, each sucrose layer was carefully removed and the nuclear pellet was suspended in 5 ml of buffer C (20 mM Tris-HCl (pH 7.5), 70 mM NaCl, 20 mM MgCl2, 3 mM CaCl2, 0.5 mM spermidine, 0.15 mM spermine and 0.5 mM dithiothreitol) and centrifuged at 2,000 g for 5 minutes at 4°C. For MNase digestion, the nuclear pellet was suspended in 100 μl of buffer C and a minimum of 15 μg of chromatin was digested with 1 U/μl MNase (N5386-50U, Sigma-Aldrich), followed by 3 minutes incubation at 37°C. The digestion was stopped by adding 18 μl of 0.5 M EDTA and 14 μl 0.1 M EGTA. The digested DNA fragments were then purified by using a QIAquick PCR purification kit (Qiagen) and subjected to a 2.5% agarose gel electrophoresis. All MNase assay experiments were repeated three times with four animals per genotype per experiment. Analysis of the relative intensity of the bands from three different chromatin digestions per each genotype was performed using the gel analyzer tool on Image J. Background was subtracted prior to the analysis of the bands. Statistical analysis was performed using 2-way Anova with Bonferroni posttests.

### Chromatin isolation from pachytene spermatocytes and MNase digestion of native chromatin to obtain a mononucleosome fraction

Chromatin from pachytene spermatocytes was obtained following the procedure described by Getun et al. [39] with small modifications. Briefly, pachytene spermatocytes were re-suspended in 1ml lysis buffer without calcium (10 mM Tris pH 7.5, 10 mM NaCl, 3 mM MgCl2, 0.4% NP-40, 0.5 mM Spermidine, 0.15 mM Spermine), incubated on ice for 10 minutes and centrifuged at 150 g for 10 minutes at 4°C. The pellet was then washed in 1ml lysis buffer with calcium (1 mM CaCl2), centrifuged at 150 *g* for 10 minutes at 4°C and re-suspended in 50μl lysis buffer with calcium. To standardize the amount of MNase necessary to obtain only a mono-nucleosome fraction, chromatin was divided in 6 equal amounts and digested with increasing concentrations of MNase (N5386-50U, Sigma-Aldrich) (0.125 U/μl, 0.25 U/μl, 0.5 U/μl, 1 U/μl, 2 U/μl, 3 U/μl), followed by 10 minutes incubation at 37°C. The digestion was stopped by adding 18 μl of 0.5 M EDTA and 14 μl 0.1M EGTA. A single mononucleosome fraction was successfully obtained by using 1 U/μl MNase. Finally, DNA from the mononucleosome fractions was extracted and purified using a QIAquick PCR purification kit (Qiagen). The concentration of the resulting DNA fragments was then quantified and used for tiled qPCR analysis.

### Tiled qPCR analysis of the sub-region of the pseudoautosomal region (PAR) hotspot

Tiled primer pairs covering the region comprising from 166,425 to 166,428 kb in the X chromosome, were designed using the PCRTiler software [59]. We obtained 50 pairs of primers with an overlap of ~50 kb that yield amplicons of ~100 kb. Primer sets are provided in S2 Table. Primer pairs were tested and those that produced multiples amplicons in the melting curves were discarded. Only primer pairs with similar amplification efficiency were used. qPCR was then performed using iTaq Universal SYBR Green Supermix (Bio-Rad Laboratories) on a CFX96 Touch Real-Time PCR Detection System (Bio-Rad Laboratories). A standard two-step real-time PCR program was used with an annealing temperature of 61°C and 40 cycles of amplification. DNA protection profiles were calculated as the fold enrichment (or protection capability) by calculating the 2-ΔCt (R value) of MNase digested input DNA Ct subtracted to the Ct of undigested genomic DNA (R = 2^-ΔCt^, ^Δ^Ct = Ct MNase treated DNA (Input)–Ct Undigested DNA (Genomic)), following the method described by Getun et al. [39].

## Supporting information

S1 Fig Depletion of BRDT induces apoptosis at late prophase I stages. Quantification of TUNEL positive spermatocytes per stages of the seminiferous tubule in WT (black bar) and *Brdt-/-* (grey bar) spermatocytes. 60 tubules per stage and per animal were counted in histological sections from three 3 months-old WT and *Brdt-/-* mice. **p<0.005, ***p<0.001.
(TIF)

S2 Fig Depletion of BRDT does not affect the global DSBs repair during zygonema and chromosome synapsis and desynapsis in zygonema and diplonema. Chromosome spreads of wild type (WT) and *Brdt-/-* zygotene (A,B; C,E) and diplotene (D,F) spermatocytes. (A-B) Immunolocalization of γH2AX (green) and SYCP3 (red). (C-F) Immunolocalization of SYCP1 (green) and SYCP3 (red).
(TIF)

S3 Fig The temporal dynamics and localization of RAD51 during prophase I is not altered by depletion of BRDT. (A) Immunolocalization of RAD51 (green) and SYCP3 (red) in WT and *Brdt-/-* spermatocytes. XY indicates the sex chromosomes. White arrow indicates the presence of RAD51 focus/foci in the PAR. (B) Number of the RAD51 foci in WT (black bars) and *Brdt-/-* (grey bars) spermatocytes. Samples were obtained from three WT and *Brdt-/-* mice. *n* = 18 and 21 zygotene, 25 and 37 early pachytene, 100 mid and late pachytene and 200 diplotene WT and *Brdt-/-* spermatocytes, respectively. *p =* 0.1. (C) Quantification of the number of spermatocytes with RAD51 foci or focus in the PAR of the sex chromosomes in late zygotene/early pachytene and mid pachytene WT (black bar) and *Brdt-/-* (grey bar) spermatocytes. Samples were obtained from three WT and *Brdt-/-* mice. *n* = 78 and 75 zygotene/early pachytene, and 85 and 89 mid pachytene WT and *Brdt-/-* spermatocytes, respectively. *p* = 0.37. Error bars indicate standard deviation.
(TIF)

S4 Fig Localization pattern of H3K4me3 is not altered by depletion of BRDT. (A-H) Immunolocalization of H3K4me3 (green) and SYCP3 (red) in WT and *Brdt-/-* spermatocytes. XY indicates the sex chromosomes. H3K4me3 signal is not observed in pachynema and early diplonema, but is readily detected in late diplotene spermatocytes throughout all the chromatin of autosome chromosomes but not in the XY. (n = 10 early pachynema, 20 mid pachynema, 20 mid and 13 late pachynema, 7 early and 10 mid/late diplonema WT and *Brdt-/-* spermatocytes, respectively per mouse, three 3 month-old WT and *Brdt-/-* mice).
(TIF)

S5 Fig Quantification of H3K4me1 signal intensity in the chromatin of autosomes and sex chromosomes in late prophase I WT (blue bars) and *Brdt-/-* (yellow bars) spermatocytes. Samples were obtained from three 3 month-old WT and *Brdt-/-* mice. n = 40 and 50 late pachytene, 30 early and 30 mid/late diplotene WT and *Brdt-/-* spermatocytes, respectively. ** *p* = 0.0021 for WT and *Brdt-/-* autosomes, ** *p* = 0.001 for WT autosomes and sex chromosomes, *** *p* = 0.0006 for WT and *Brdt-/-* sex chromosomes.
(TIF)

S6 Fig Lack of BRDT produces a different pattern of mis-regulation of gene expression in autosomes versus the X chromosome. (A) RNA seq analysis per chromosome of 20dpp *Brdt-/-* and WT testis. Results show the number of upregulated (green bars) and downregulated (red bars) genes with an adjusted p-value of ≤0.05 and an absolute fold-change of <1.5. (B) Quantitative reverse transcriptase PCR (qRT-PCR) analysis of representative X-linked genes in WT (black bars) and *Brdt-/-* (grey bars) enriched spermatocyte fractions. The elevated expression of all genes except *Ddx3x* was confirmed. There is a significant increase in the expression of *Nxf2*, *Fmr1*, *Gm5072 and Huwe1*. Samples were obtained from seven WT and sixteen *Brdt-/-* 2–3 month-old mice. Triplicates per each reaction were performed. Error bars indicate standard deviation. * *p*<0.05, *** *p*<0.001.
(TIF)

S7 Fig Gene expression analysis of genes that encode proteins involved in CO formation and maturation. qPCR analysis of the expression of CO protein-related genes in WT (black bars) and *Brdt-/-* (grey bars) spermatocytes. *p* = 0.53. Error bars indicate standard deviation. Samples were obtained from seven WT and sixteen *Brdt-/-* 2–3 month-old mice. Triplicates for each reaction were performed.
(TIF)

S1 Table Primer sets used for gene expression analysis in WT and *Brdt-/-* spermatocytes. (PDF)

S2 Table Primer sets used for tiled qPCR analysis of the sub-region of the PAR hotspot. (PDF)

S3 Table Expression profile of genes in autosomes and X chromosomes in 17dpp and 20dpp *Brdt-/-* testes. (XLSX)

## References

[1] Berkovits BD, Wolgemuth DJ. The role of the double bromodomain-containing BET genes during mammalian spermatogenesis. Curr Top Dev Biol. 2013;102:293–326. Epub 2013/01/05. doi: 10.1016/B978-0-12-416024-8.00011-8 . 23287038 10.1016/B978-0-12-416024-8.00011-8 PMC3918955

[2] Shi J, Vakoc CR. The mechanisms behind the therapeutic activity of BET bromodomain inhibition. Molecular cell. 2014;54(5):728–36. doi: 10.1016/j.molcel.2014.05.016 ; PubMed Central PMCID: PMC4236231. 24905006 10.1016/j.molcel.2014.05.016 PMC4236231

[3] Wang CY, Filippakopoulos P. Beating the odds: BETs in disease. Trends in biochemical sciences. 2015;40(8):468–79. doi: 10.1016/j.tibs.2015.06.002 . 26145250 10.1016/j.tibs.2015.06.002

[4] Filippakopoulos P, Knapp S. Targeting bromodomains: epigenetic readers of lysine acetylation. Nature reviews Drug discovery. 2014;13(5):337–56. doi: 10.1038/nrd4286 . 24751816 10.1038/nrd4286

[5] Shang E, Salazar G, Crowley TE, Wang X, Lopez RA, Wang X, et al Identification of unique, differentiation stage-specific patterns of expression of the bromodomain-containing genes Brd2, Brd3, Brd4, and Brdt in the mouse testis. Gene expression patterns: GEP. 2004;4(5):513–9. doi: 10.1016/j.modgep.2004.03.002 . 15261828 10.1016/j.modgep.2004.03.002

[6] Shang E, Nickerson HD, Wen D, Wang X, Wolgemuth DJ. The first bromodomain of Brdt, a testis-specific member of the BET sub-family of double-bromodomain-containing proteins, is essential for male germ cell differentiation. Development. 2007;134(19):3507–15. doi: 10.1242/dev.004481 . 17728347 10.1242/dev.004481

[7] Berkovits BD, Wolgemuth DJ. The first bromodomain of the testis-specific double bromodomain protein Brdt is required for chromocenter organization that is modulated by genetic background. Developmental biology. 2011;360(2):358–68. Epub 2011/10/25. doi: 10.1016/j.ydbio.2011.10.005 ; PubMed Central PMCID: PMC3217133. 22020252 10.1016/j.ydbio.2011.10.005 PMC3217133

[8] Gaucher J, Boussouar F, Montellier E, Curtet S, Buchou T, Bertrand S, et al Bromodomain-dependent stage-specific male genome programming by Brdt. The EMBO journal. 2012;31(19):3809–20. doi: 10.1038/emboj.2012.233 ; PubMed Central PMCID: PMC3463845. 22922464 10.1038/emboj.2012.233 PMC3463845

[9] Barda S, Paz G, Yogev L, Yavetz H, Lehavi O, Hauser R, et al Expression of BET genes in testis of men with different spermatogenic impairments. Fertility and sterility. 2012;97(1):46–52 e5. doi: 10.1016/j.fertnstert.2011.10.010 . 22035730 10.1016/j.fertnstert.2011.10.010

[10] Li L, Sha Y, Wang X, Li P, Wang J, Kee K, et al Whole-exome sequencing identified a homozygous BRDT mutation in a patient with acephalic spermatozoa. Oncotarget. 2017 doi: 10.18632/oncotarget.15251 . 28199965 10.18632/oncotarget.15251 PMC5386733

[11] Matzuk MM, McKeown MR, Filippakopoulos P, Li Q, Ma L, Agno JE, et al Small-molecule inhibition of BRDT for male contraception. Cell. 2012;150(4):673–84. doi: 10.1016/j.cell.2012.06.045 ; PubMed Central PMCID: PMC3420011. 22901802 10.1016/j.cell.2012.06.045 PMC3420011

[12] Miller TC, Simon B, Rybin V, Grotsch H, Curtet S, Khochbin S, et al A bromodomain-DNA interaction facilitates acetylation-dependent bivalent nucleosome recognition by the BET protein BRDT. Nature communications. 2016;7:13855 doi: 10.1038/ncomms13855 ; PubMed Central PMCID: PMC5187433. 27991587 10.1038/ncomms13855 PMC5187433

[13] Berkovits BD, Wang L, Guarnieri P, Wolgemuth DJ. The testis-specific double bromodomain-containing protein BRDT forms a complex with multiple spliceosome components and is required for mRNA splicing and 3'-UTR truncation in round spermatids. Nucleic acids research. 2012;40(15):7162–75. Epub 2012/05/10. doi: 10.1093/nar/gks342 PubMed Central PMCID: PMC3424537. 22570411 10.1093/nar/gks342 PMC3424537

[14] Wang L, Wolgemuth DJ. BET Protein BRDT Complexes With HDAC1, PRMT5, and TRIM28 and Functions in Transcriptional Repression During Spermatogenesis. Journal of cellular biochemistry. 2016;117(6):1429–38. doi: 10.1002/jcb.25433 ; PubMed Central PMCID: PMC4916496. 26565999 10.1002/jcb.25433 PMC4916496

[15] Crichton JH, Playfoot CJ, Adams IR. The role of chromatin modifications in progression through mouse meiotic prophase. Journal of genetics and genomics = Yi chuan xue bao. 2014;41(3):97–106. doi: 10.1016/j.jgg.2014.01.003 . 24656230 10.1016/j.jgg.2014.01.003

[16] Kota SK, Feil R. Epigenetic transitions in germ cell development and meiosis. Developmental cell. 2010;19(5):675–86. doi: 10.1016/j.devcel.2010.10.009 . 21074718 10.1016/j.devcel.2010.10.009

[17] Borde V, Robine N, Lin W, Bonfils S, Geli V, Nicolas A. Histone H3 lysine 4 trimethylation marks meiotic recombination initiation sites. The EMBO journal. 2009;28(2):99–111. doi: 10.1038/emboj.2008.257 ; PubMed Central PMCID: PMC2634730. 19078966 10.1038/emboj.2008.257 PMC2634730

[18] Hu J, Donahue G, Dorsey J, Govin J, Yuan Z, Garcia BA, et al H4K44 Acetylation Facilitates Chromatin Accessibility during Meiosis. Cell reports. 2015;13(9):1772–80. doi: 10.1016/j.celrep.2015.10.070 ; PubMed Central PMCID: PMC4793274. 26628362 10.1016/j.celrep.2015.10.070 PMC4793274

[19] Kobayashi W, Takaku M, Machida S, Tachiwana H, Maehara K, Ohkawa Y, et al Chromatin architecture may dictate the target site for DMC1, but not for RAD51, during homologous pairing. Scientific reports. 2016;6:24228 doi: 10.1038/srep24228 ; PubMed Central PMCID: PMC4823753. 27052786 10.1038/srep24228 PMC4823753

[20] Pidoux AL, Allshire RC. The role of heterochromatin in centromere function. Philosophical transactions of the Royal Society of London Series B, Biological sciences. 2005;360(1455):569–79. doi: 10.1098/rstb.2004.1611 ; PubMed Central PMCID: PMC1569473. 15905142 10.1098/rstb.2004.1611 PMC1569473

[21] van der Heijden GW, Derijck AA, Posfai E, Giele M, Pelczar P, Ramos L, et al Chromosome-wide nucleosome replacement and H3.3 incorporation during mammalian meiotic sex chromosome inactivation. Nature genetics. 2007;39(2):251–8. doi: 10.1038/ng1949 . 17237782 10.1038/ng1949

[22] Baarends WM, Wassenaar E, van der Laan R, Hoogerbrugge J, Sleddens-Linkels E, Hoeijmakers JH, et al Silencing of unpaired chromatin and histone H2A ubiquitination in mammalian meiosis. Molecular and cellular biology. 2005;25(3):1041–53. doi: 10.1128/MCB.25.3.1041-1053.2005 ; PubMed Central PMCID: PMC543997. 15657431 10.1128/MCB.25.3.1041-1053.2005 PMC543997

[23] Luo M, Zhou J, Leu NA, Abreu CM, Wang J, Anguera MC, et al Polycomb protein SCML2 associates with USP7 and counteracts histone H2A ubiquitination in the XY chromatin during male meiosis. PLoS genetics. 2015;11(1):e1004954 doi: 10.1371/journal.pgen.1004954 ; PubMed Central PMCID: PMC4310598. 25634095 10.1371/journal.pgen.1004954 PMC4310598

[24] Manterola M, Page J, Vasco C, Berrios S, Parra MT, Viera A, et al A high incidence of meiotic silencing of unsynapsed chromatin is not associated with substantial pachytene loss in heterozygous male mice carrying multiple simple robertsonian translocations. PLoS genetics. 2009;5(8):e1000625 Epub 2009/08/29. doi: 10.1371/journal.pgen.1000625 ; PubMed Central PMCID: PMC2726437. 19714216 10.1371/journal.pgen.1000625 PMC2726437

[25] Prigent C, Dimitrov S. Phosphorylation of serine 10 in histone H3, what for? Journal of cell science. 2003;116(Pt 18):3677–85. doi: 10.1242/jcs.00735 . 12917355 10.1242/jcs.00735

[26] Kauppi L, Barchi M, Baudat F, Romanienko PJ, Keeney S, Jasin M. Distinct properties of the XY pseudoautosomal region crucial for male meiosis. Science. 2011;331(6019):916–20. doi: 10.1126/science.1195774 ; PubMed Central PMCID: PMC3151169. 21330546 10.1126/science.1195774 PMC3151169

[27] Dai J, Voloshin O, Potapova S, Camerini-Otero RD. Meiotic Knockdown and Complementation Reveals Essential Role of RAD51 in Mouse Spermatogenesis. Cell reports. 2017;18(6):1383–94. doi: 10.1016/j.celrep.2017.01.024 . 28178517 10.1016/j.celrep.2017.01.024 PMC5358547

[28] Page J, de la Fuente R, Manterola M, Parra MT, Viera A, Berrios S, et al Inactivation or non-reactivation: what accounts better for the silence of sex chromosomes during mammalian male meiosis? Chromosoma. 2012;121(3):307–26. doi: 10.1007/s00412-012-0364-y . 22366883 10.1007/s00412-012-0364-y

[29] Cheng J, Blum R, Bowman C, Hu D, Shilatifard A, Shen S, et al A role for H3K4 monomethylation in gene repression and partitioning of chromatin readers. Molecular cell. 2014;53(6):979–92. doi: 10.1016/j.molcel.2014.02.032 ; PubMed Central PMCID: PMC4031464. 24656132 10.1016/j.molcel.2014.02.032 PMC4031464

[30] Schultz N, Hamra FK, Garbers DL. A multitude of genes expressed solely in meiotic or postmeiotic spermatogenic cells offers a myriad of contraceptive targets. Proceedings of the National Academy of Sciences of the United States of America. 2003;100(21):12201–6. doi: 10.1073/pnas.1635054100 ; PubMed Central PMCID: PMC218736. 14526100 10.1073/pnas.1635054100 PMC218736

[31] Kleckner N, Storlazzi A, Zickler D. Coordinate variation in meiotic pachytene SC length and total crossover/chiasma frequency under conditions of constant DNA length. Trends in genetics: TIG. 2003;19(11):623–8. doi: 10.1016/j.tig.2003.09.004 . 14585614 10.1016/j.tig.2003.09.004

[32] Novak I, Wang H, Revenkova E, Jessberger R, Scherthan H, Hoog C. Cohesin Smc1beta determines meiotic chromatin axis loop organization. The Journal of cell biology. 2008;180(1):83–90. doi: 10.1083/jcb.200706136 ; PubMed Central PMCID: PMC2213612. 18180366 10.1083/jcb.200706136 PMC2213612

[33] Mezard C, Jahns MT, Grelon M. Where to cross? New insights into the location of meiotic crossovers. Trends in genetics: TIG. 2015;31(7):393–401. doi: 10.1016/j.tig.2015.03.008 . 25907025 10.1016/j.tig.2015.03.008

[34] Kauppi L, Jasin M, Keeney S. The tricky path to recombining X and Y chromosomes in meiosis. Annals of the New York Academy of Sciences. 2012;1267:18–23. doi: 10.1111/j.1749-6632.2012.06593.x ; PubMed Central PMCID: PMC3631422. 22954211 10.1111/j.1749-6632.2012.06593.x PMC3631422

[35] Anderson LK, Reeves A, Webb LM, Ashley T. Distribution of crossing over on mouse synaptonemal complexes using immunofluorescent localization of MLH1 protein. Genetics. 1999;151(4):1569–79. ; PubMed Central PMCID: PMC1460565. 10101178 10.1093/genetics/151.4.1569 PMC1460565

[36] Su Y, Barton AB, Kaback DB. Decreased meiotic reciprocal recombination in subtelomeric regions in Saccharomyces cerevisiae. Chromosoma. 2000;109(7):467–75. . 11151676 10.1007/s004120000098

[37] Talbert PB, Henikoff S. Centromeres convert but don't cross. PLoS biology. 2010;8(3):e1000326 doi: 10.1371/journal.pbio.1000326 ; PubMed Central PMCID: PMC2834710. 20231873 10.1371/journal.pbio.1000326 PMC2834710

[38] Smagulova F, Gregoretti IV, Brick K, Khil P, Camerini-Otero RD, Petukhova GV. Genome-wide analysis reveals novel molecular features of mouse recombination hotspots. Nature. 2011;472(7343):375–8. doi: 10.1038/nature09869 ; PubMed Central PMCID: PMC3117304. 21460839 10.1038/nature09869 PMC3117304

[39] Getun IV, Wu ZK, Khalil AM, Bois PR. Nucleosome occupancy landscape and dynamics at mouse recombination hotspots. EMBO reports. 2010;11(7):555–60. doi: 10.1038/embor.2010.79 ; PubMed Central PMCID: PMC2897116. 20508641 10.1038/embor.2010.79 PMC2897116

[40] Dey A, Chitsaz F, Abbasi A, Misteli T, Ozato K. The double bromodomain protein Brd4 binds to acetylated chromatin during interphase and mitosis. Proceedings of the National Academy of Sciences of the United States of America. 2003;100(15):8758–63. doi: 10.1073/pnas.1433065100 ; PubMed Central PMCID: PMC166386. 12840145 10.1073/pnas.1433065100 PMC166386

[41] Umehara T, Nakamura Y, Jang MK, Nakano K, Tanaka A, Ozato K, et al Structural basis for acetylated histone H4 recognition by the human BRD2 bromodomain. The Journal of biological chemistry. 2010;285(10):7610–8. doi: 10.1074/jbc.M109.062422 ; PubMed Central PMCID: PMC2844208. 20048151 10.1074/jbc.M109.062422 PMC2844208

[42] Vernet N, Mahadevaiah SK, de Rooij DG, Burgoyne PS, Ellis PJ. Zfy genes are required for efficient meiotic sex chromosome inactivation (MSCI) in spermatocytes. Human molecular genetics. 2016 doi: 10.1093/hmg/ddw344 . 27742779 10.1093/hmg/ddw344 PMC5418838

[43] Zheng K, Yang F, Wang PJ. Regulation of male fertility by X-linked genes. Journal of andrology. 2010;31(1):79–85. doi: 10.2164/jandrol.109.008193 ; PubMed Central PMCID: PMC2931805. 19875494 10.2164/jandrol.109.008193 PMC2931805

[44] Pan J, Eckardt S, Leu NA, Buffone MG, Zhou J, Gerton GL, et al Inactivation of Nxf2 causes defects in male meiosis and age-dependent depletion of spermatogonia. Developmental biology. 2009;330(1):167–74. doi: 10.1016/j.ydbio.2009.03.022 ; PubMed Central PMCID: PMC2702087. 19345203 10.1016/j.ydbio.2009.03.022 PMC2702087

[45] Yang F, Gell K, van der Heijden GW, Eckardt S, Leu NA, Page DC, et al Meiotic failure in male mice lacking an X-linked factor. Genes & development. 2008;22(5):682–91. doi: 10.1101/gad.1613608 ; PubMed Central PMCID: PMC2259036. 18316482 10.1101/gad.1613608 PMC2259036

[46] Zickler D, Kleckner N. Meiotic chromosomes: integrating structure and function. Annual review of genetics. 1999;33:603–754. doi: 10.1146/annurev.genet.33.1.603 . 10690419 10.1146/annurev.genet.33.1.603

[47] Yelina NE, Lambing C, Hardcastle TJ, Zhao X, Santos B, Henderson IR. DNA methylation epigenetically silences crossover hot spots and controls chromosomal domains of meiotic recombination in Arabidopsis. Genes & development. 2015;29(20):2183–202. doi: 10.1101/gad.270876.115 ; PubMed Central PMCID: PMC4617981. 26494791 10.1101/gad.270876.115 PMC4617981

[48] Carlton PM, Farruggio AP, Dernburg AF. A link between meiotic prophase progression and crossover control. PLoS genetics. 2006;2(2):e12 doi: 10.1371/journal.pgen.0020012 ; PubMed Central PMCID: PMC1359072. 16462941 10.1371/journal.pgen.0020012 PMC1359072

[49] Hunter N. Meiotic Recombination: The Essence of Heredity. Cold Spring Harbor perspectives in biology. 2015;7(12). doi: 10.1101/cshperspect.a016618 . 26511629 10.1101/cshperspect.a016618 PMC4665078

[50] Zhang L, Wang S, Yin S, Hong S, Kim KP, Kleckner N. Topoisomerase II mediates meiotic crossover interference. Nature. 2014;511(7511):551–6. doi: 10.1038/nature13442 ; PubMed Central PMCID: PMC4128387. 25043020 10.1038/nature13442 PMC4128387

[51] Hayashi M, Mlynarczyk-Evans S, Villeneuve AM. The synaptonemal complex shapes the crossover landscape through cooperative assembly, crossover promotion and crossover inhibition during Caenorhabditis elegans meiosis. Genetics. 2010;186(1):45–58. doi: 10.1534/genetics.110.115501 ; PubMed Central PMCID: PMC2940310. 20592266 10.1534/genetics.110.115501 PMC2940310

[52] Shinohara M, Oh SD, Hunter N, Shinohara A. Crossover assurance and crossover interference are distinctly regulated by the ZMM proteins during yeast meiosis. Nature genetics. 2008;40(3):299–309. doi: 10.1038/ng.83 . 18297071 10.1038/ng.83

[53] Lynn A, Schrump S, Cherry J, Hassold T, Hunt P. Sex, not genotype, determines recombination levels in mice. American journal of human genetics. 2005;77(4):670–5. doi: 10.1086/491718 ; PubMed Central PMCID: PMC1275616. 16175513 10.1086/491718 PMC1275616

[54] Skarnes WC, Rosen B, West AP, Koutsourakis M, Bushell W, Iyer V, et al A conditional knockout resource for the genome-wide study of mouse gene function. Nature. 2011;474(7351):337–42. doi: 10.1038/nature10163 ; PubMed Central PMCID: PMC3572410. 21677750 10.1038/nature10163 PMC3572410

[55] Chung SS, Wang X, Wolgemuth DJ. Male sterility in mice lacking retinoic acid receptor alpha involves specific abnormalities in spermiogenesis. Differentiation; research in biological diversity. 2005;73(4):188–98. doi: 10.1111/j.1432-0436.2005.00018.x ; PubMed Central PMCID: PMC3785313. 15901285 10.1111/j.1432-0436.2005.00018.x PMC3785313

[56] Ayarza E, Gonzalez M, Lopez F, Fernandez-Donoso R, Page J, Berrios S. Alterations in chromosomal synapses and DNA repair in apoptotic spermatocytes of Mus m. domesticus. European journal of histochemistry: EJH. 2016;60(2):2677 doi: 10.4081/ejh.2016.2677 ; PubMed Central PMCID: PMC4933834. 27349323 10.4081/ejh.2016.2677 PMC4933834

[57] Martinerie L, Manterola M, Chung SS, Panigrahi SK, Weisbach M, Vasileva A, et al Mammalian E-type cyclins control chromosome pairing, telomere stability and CDK2 localization in male meiosis. PLoS genetics. 2014;10(2):e1004165 doi: 10.1371/journal.pgen.1004165 ; PubMed Central PMCID: PMC3937215. 24586195 10.1371/journal.pgen.1004165 PMC3937215

[58] Kishi Y, Fujii Y, Hirabayashi Y, Gotoh Y. HMGA regulates the global chromatin state and neurogenic potential in neocortical precursor cells. Nature neuroscience. 2012;15(8):1127–33. doi: 10.1038/nn.3165 . 22797695 10.1038/nn.3165

[59] Gervais AL, Marques M, Gaudreau L. PCRTiler: automated design of tiled and specific PCR primer pairs. Nucleic acids research. 2010;38(Web Server issue):W308–12. doi: 10.1093/nar/gkq485 ; PubMed Central PMCID: PMC2896098. 20519202 10.1093/nar/gkq485 PMC2896098

