# Supplementary figures and images for: BRDT is an essential epigenetic regulator for proper chromatin organization, silencing of sex chromosomes and crossover formation in male meiosis
Source: PLoS Genet. 2018 Mar 7;14(3):e1007209. doi: 10.1371/journal.pgen.1007209 (PMC5841650; doi:10.1371/journal.pgen.1007209)

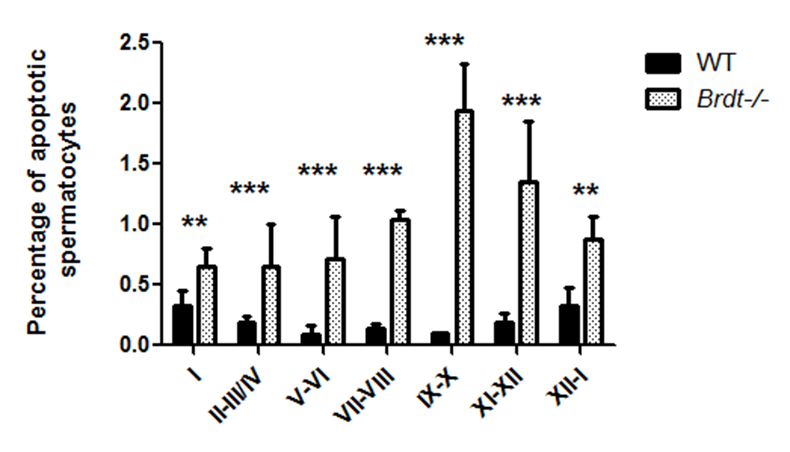

Supplement: S1 Fig — Quantification of TUNEL positive spermatocytes per stages of the seminiferous tubule in WT (black bar) and Brdt-/- (grey bar) spermatocytes. 60 tubules per stage and per animal were counted in histological sections from three 3 months-old WT and Brdt-/- mice. **p<0.005, ***p<0.001. (TIF) [file pgen.1007209.s001.tif]

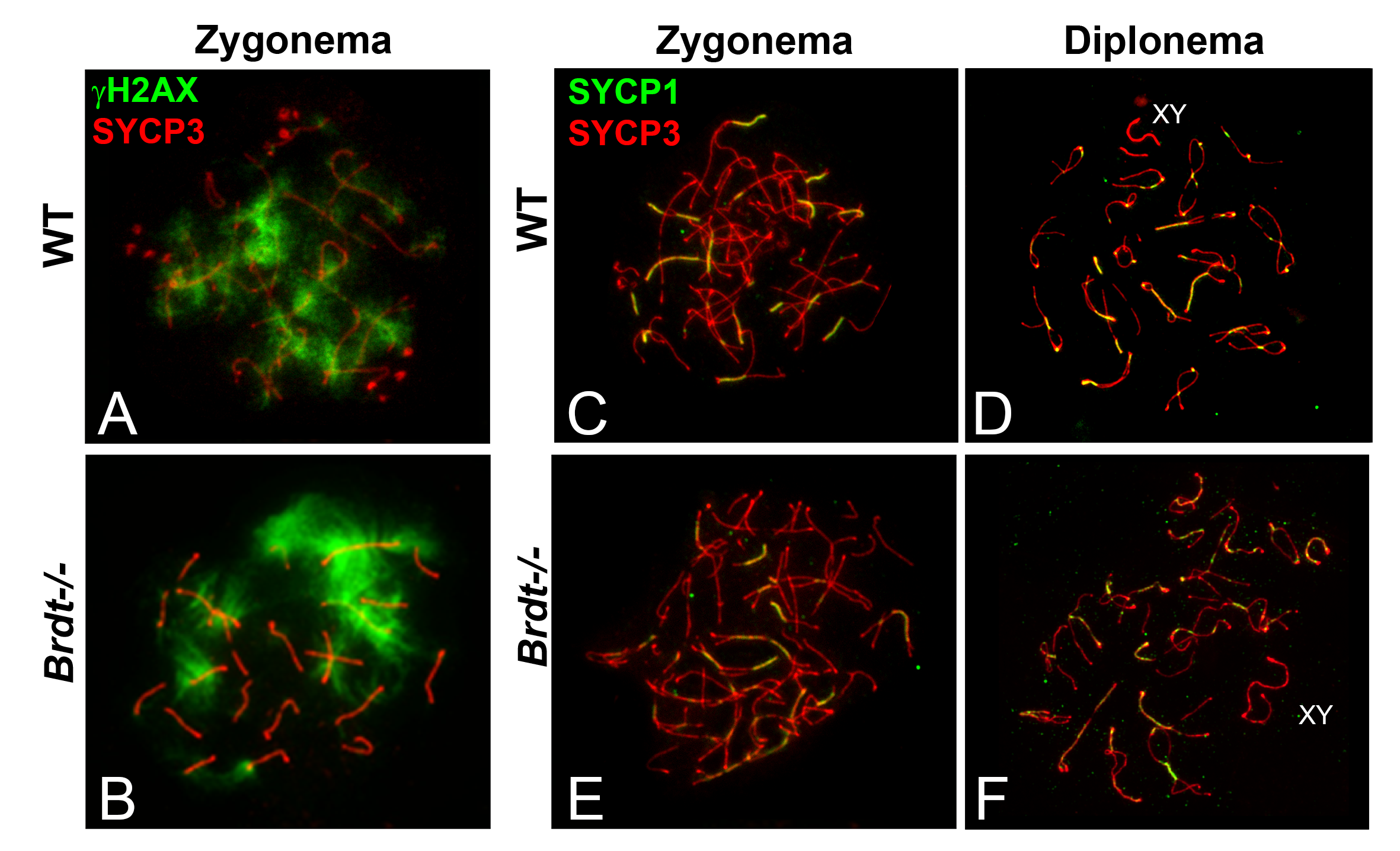

Supplement: S2 Fig — Chromosome spreads of wild type (WT) and Brdt-/- zygotene (A,B; C,E) and diplotene (D,F) spermatocytes. (A-B) Immunolocalization of γH2AX (green) and SYCP3 (red). (C-F) Immunolocalization of SYCP1 (green) and SYCP3 (red). (TIF) [file pgen.1007209.s002.tif]

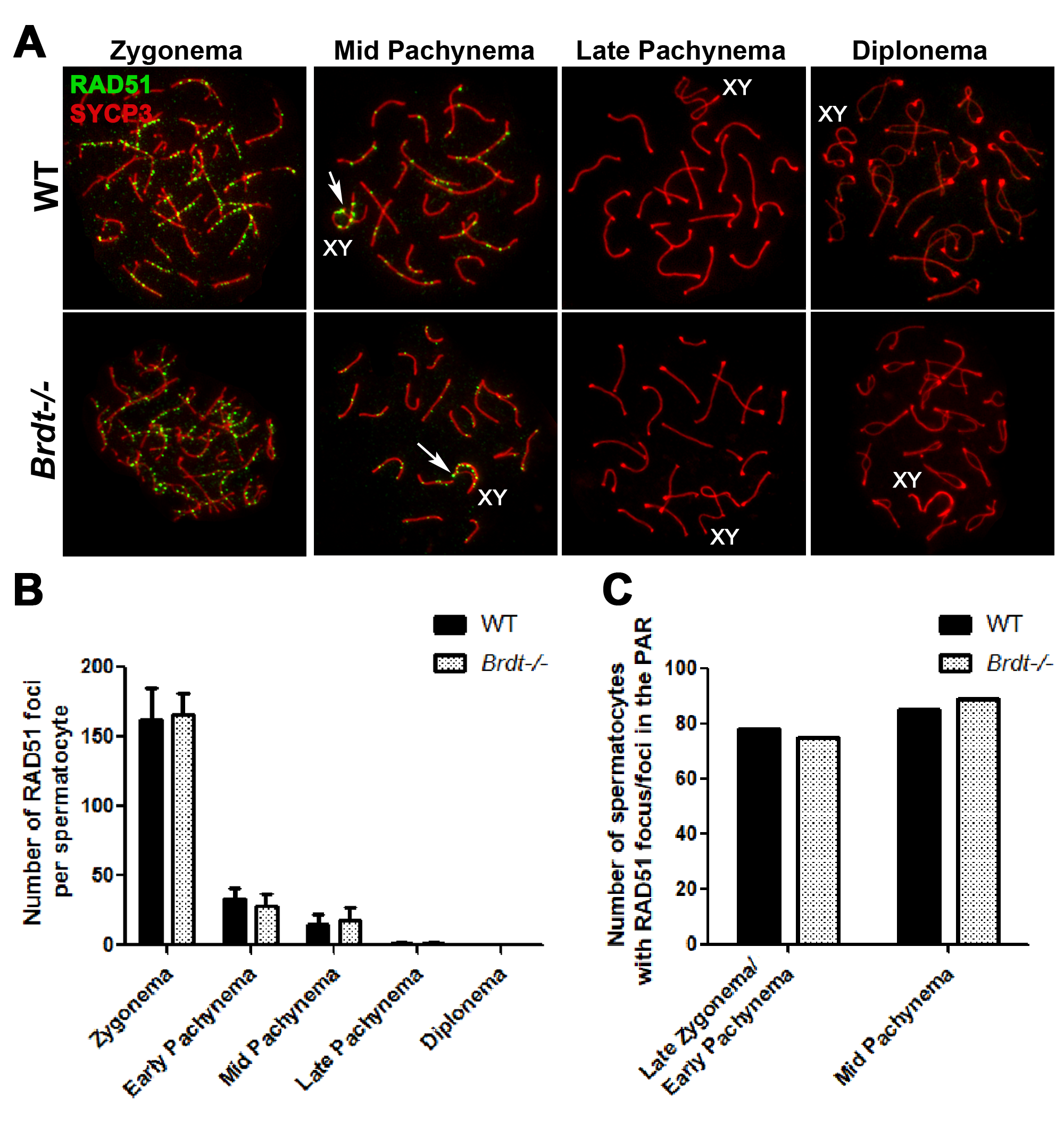

Supplement: S3 Fig — (A) Immunolocalization of RAD51 (green) and SYCP3 (red) in WT and Brdt-/- spermatocytes. XY indicates the sex chromosomes. White arrow indicates the presence of RAD51 focus/foci in the PAR. (B) Number of the RAD51 foci in WT (black bars) and Brdt-/- (grey bars) spermatocytes. Samples were obtained from three WT and Brdt-/- mice. n = 18 and 21 zygotene, 25 and 37 early pachytene, 100 mid and late pachytene and 200 diplotene WT and Brdt-/- spermatocytes, respectively. p = 0.1. (C) Quantification of the number of spermatocytes with RAD51 foci or focus in the PAR of the sex chromosomes in late zygotene/early pachytene and mid pachytene WT (black bar) and Brdt-/- (grey bar) spermatocytes. Samples were obtained from three WT and Brdt-/- mice. n = 78 and 75 zygotene/early pachytene, and 85 and 89 mid pachytene WT and Brdt-/- spermatocytes, respectively. p = 0.37. Error bars indicate standard deviation. (TIF) [file pgen.1007209.s003.tif]

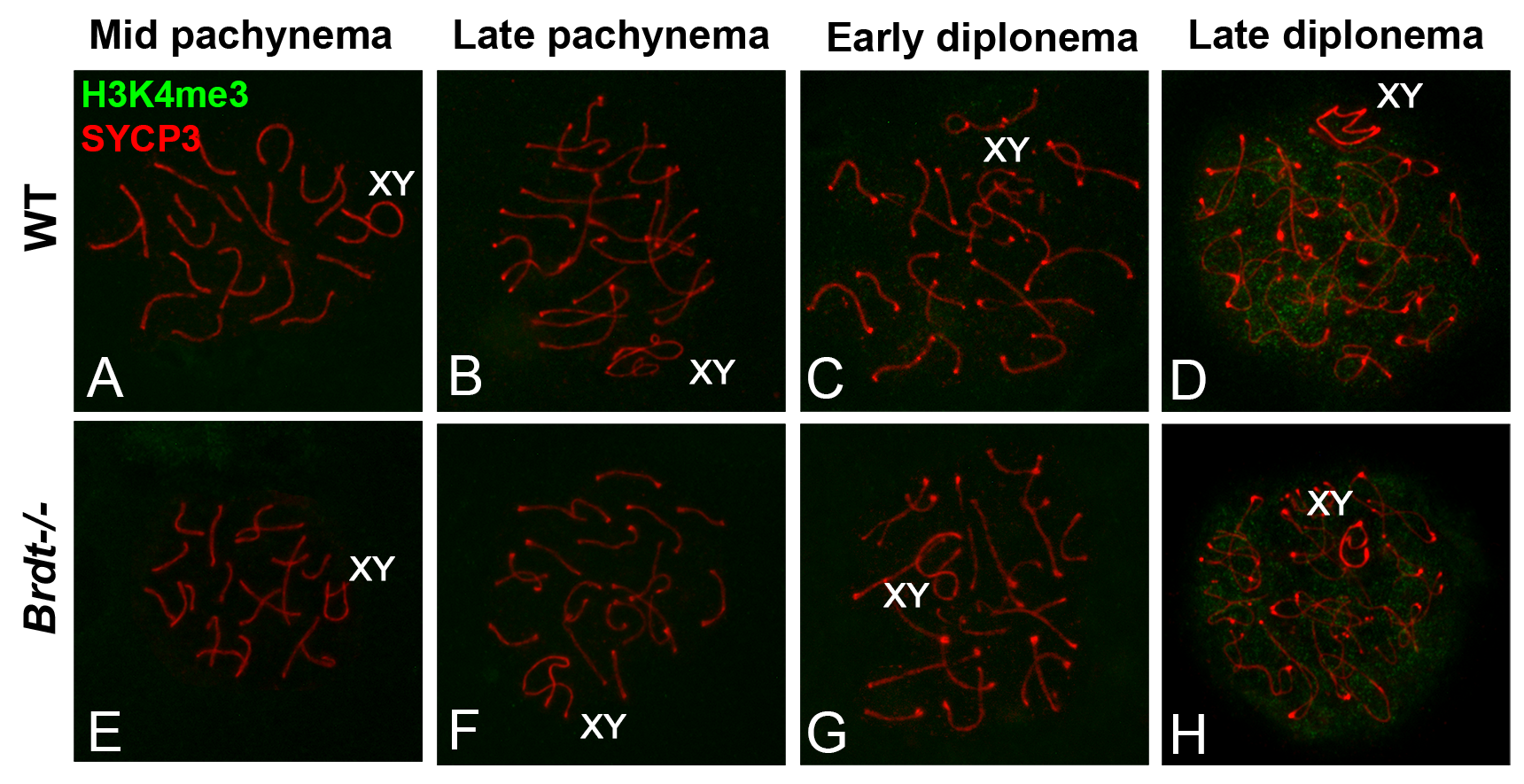

Supplement: S4 Fig — (A-H) Immunolocalization of H3K4me3 (green) and SYCP3 (red) in WT and Brdt-/- spermatocytes. XY indicates the sex chromosomes. H3K4me3 signal is not observed in pachynema and early diplonema, but is readily detected in late diplotene spermatocytes throughout all the chromatin of autosome chromosomes but not in the XY. (n = 10 early pachynema, 20 mid pachynema, 20 mid and 13 late pachynema, 7 early and 10 mid/late diplonema WT and Brdt-/- spermatocytes, respectively per mouse, three 3 month-old WT and Brdt-/- mice). (TIF) [file pgen.1007209.s004.tif]

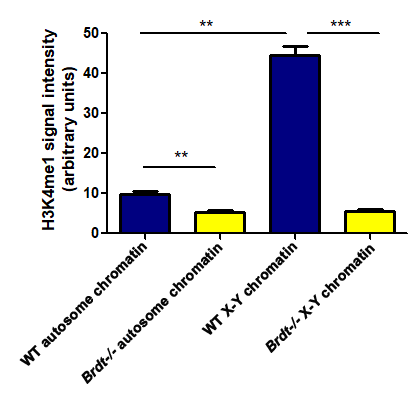

Supplement: S5 Fig — Samples were obtained from three 3 month-old WT and Brdt-/- mice. n = 40 and 50 late pachytene, 30 early and 30 mid/late diplotene WT and Brdt-/- spermatocytes, respectively. ** p = 0.0021 for WT and Brdt-/- autosomes, ** p = 0.001 for WT autosomes and sex chromosomes, *** p = 0.0006 for WT and Brdt-/- sex chromosomes. (TIF) [file pgen.1007209.s005.tif]

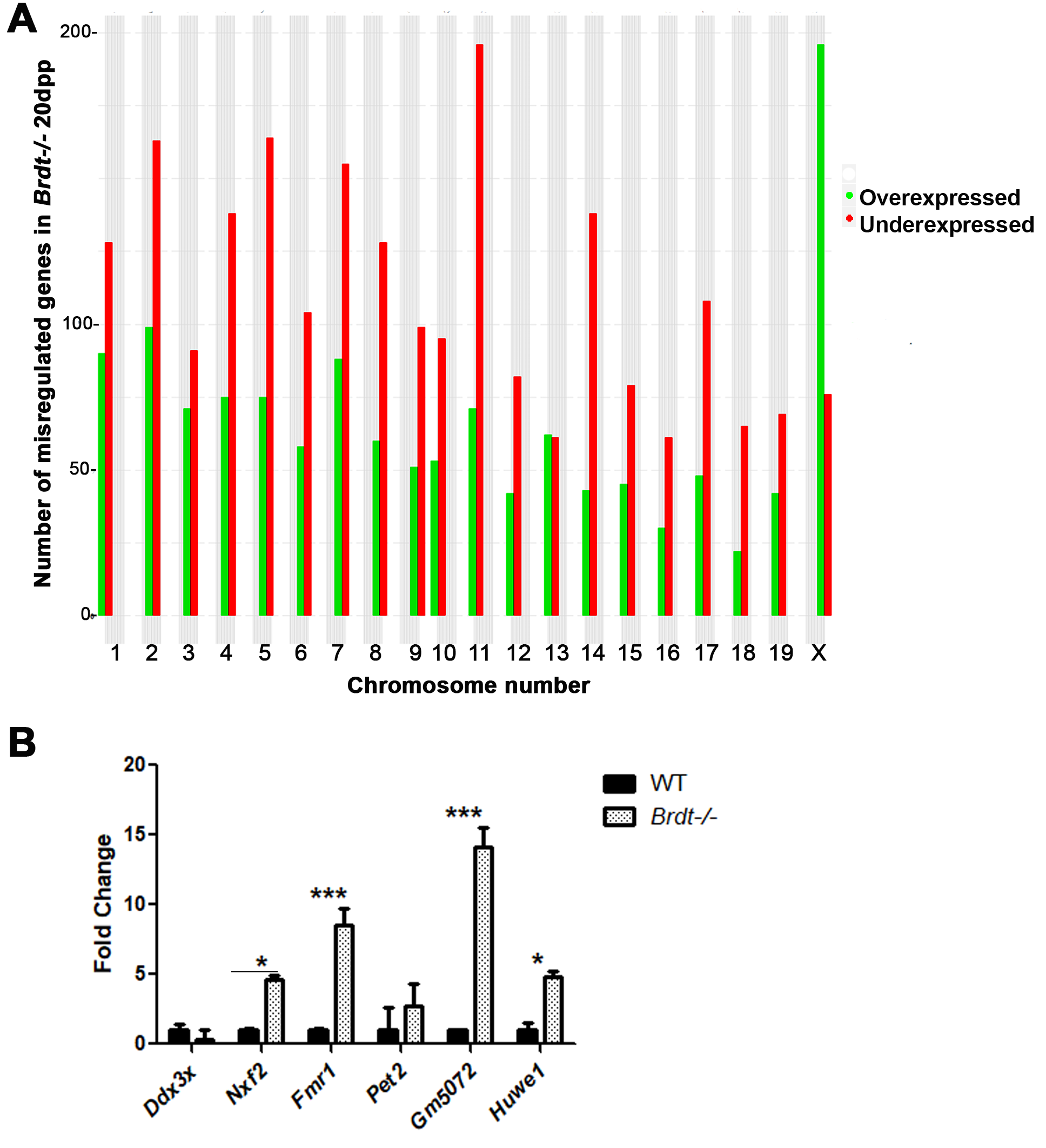

Supplement: S6 Fig — (A) RNA seq analysis per chromosome of 20dpp Brdt-/- and WT testis. Results show the number of upregulated (green bars) and downregulated (red bars) genes with an adjusted p-value of ≤0.05 and an absolute fold-change of <1.5. (B) Quantitative reverse transcriptase PCR (qRT-PCR) analysis of representative X-linked genes in WT (black bars) and Brdt-/- (grey bars) enriched spermatocyte fractions. The elevated expression of all genes except Ddx3x was confirmed. There is a significant increase in the expression of Nxf2, Fmr1, Gm5072 and Huwe1. Samples were obtained from seven WT and sixteen Brdt-/- 2–3 month-old mice. Triplicates per each reaction were performed. Error bars indicate standard deviation. * p<0.05, *** p<0.001. (TIF) [file pgen.1007209.s006.tif]

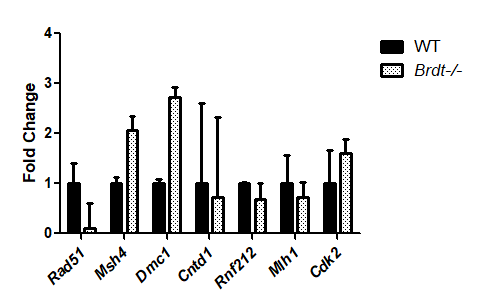

Supplement: S7 Fig — qPCR analysis of the expression of CO protein-related genes in WT (black bars) and Brdt-/- (grey bars) spermatocytes. p = 0.53. Error bars indicate standard deviation. Samples were obtained from seven WT and sixteen Brdt-/- 2–3 month-old mice. Triplicates for each reaction were performed. (TIF) [file pgen.1007209.s007.tif]
