# Supplementary material for: BRDT is an essential epigenetic regulator for proper chromatin organization, silencing of sex chromosomes and crossover formation in male meiosis
Source: PLoS Genet. 2018 Mar 7;14(3):e1007209. doi: 10.1371/journal.pgen.1007209 (PMC5841650; doi:10.1371/journal.pgen.1007209)
Supplement: S1 Table — (PDF) [file pgen.1007209.s008.pdf]

S1 table

Primer sets used for gene expression analysis in WT and *Brdt-/-* spermatocytes

Gene	Primer Forward	Primer Reverse	Reference
Cdkl5	CTGAGCAGATGAAGCTTTTCTACAGT	TGAGGATGGTTAACAGCTGGAA	[1]
Ddx3x	ATGAGTCATGTGGCAGTGGA	GGTGGGATATAACGCCCTTT	--
Pet2	TGACGATGACGACGAAGAAG	CCCAGACTCTGCTCTTCCAC	--
Scml2	ATCTTCCCAGTTGGATGGTG	CTGGGGCCTCTTCTTCATTT	[2]
Nxf2	GCCTGCTTCTCCTTGTCA	GCCCTCATTCTGGAGAGC	[3]
Fmr1	GCAGATTCCATTTCATGATGTCA	ACCACCAACAGCAAGGCTCT	[4]
Gm5072	GCCACTGAGGAAGAGGTCTG	GGGACCCCATAGGAACTCAT	--
Huwe1	CCAGCCATCTCCTAGTGCTC	CCCTAGCATGTCCCACAGTT	--
Mlh1	TGCTGGCCTTAGACAGTCCT	CAATCAGGTTCCCTTCCTCA	--
Cdk2	CCCCAGAACCTGCTTATCAA	GCAGCCCAGAAGAATTTCAG	[5]
Rad51	AGCCGGTTACCATACAGTGG	GTGGTGAAACCCATTGGAAC	--
Msh4	TCTACGAGCACATCCACTGC	GCCATCCTTGTTTGATTGCT	--
Dmc1	CCCAGATGTTGTCACGACTC	AGCGGTTATTGCGAAGGTGG	[6]
Cntd1	CGGATCGTGGAGTTTGTTTT	CAGCTCTGCAGCTCTGTGTC	--
Rnf212	ATCATCACAACAACCAGCTTTCAAC	TAGTTACATCATCTCTCTAGAGTG	--
Arbp	CAAAGCTGAAGCAAAGGAAGAG	AATTAAGCAGGCTGACTTGGTTG	[7]

1. Mari F, Azimonti S, Bertani I, Bolognese F, Colombo E, Caselli R, et al. CDKL5 belongs to the same molecular pathway of MeCP2 and it is responsible for the early-onset seizure variant of Rett syndrome. Human molecular genetics. 2005;14(14):1935-46. doi: 10.1093/hmg/ddi198. PubMed PMID: 15917271.

2. Branco MR, King M, Perez-Garcia V, Bogutz AB, Caley M, Fineberg E, et al. Maternal DNA Methylation Regulates Early Trophoblast Development. Developmental cell. 2016;36(2):152-63. doi: 10.1016/j.devcel.2015.12.027. PubMed PMID: 26812015; PubMed Central PMCID: PMC4729543.

3. Zhang M, Wang Q, Huang Y. Fragile X mental retardation protein FMRP and the RNA export factor NXF2 associate with and destabilize Nxf1 mRNA in neuronal cells. Proceedings of the National Academy of Sciences of the United States of America. 2007;104(24):10057-62. doi: 10.1073/pnas.0700169104. PubMed PMID: 17548835; PubMed Central PMCID: PMC1891223.

4. Tassone F, Hagerman RJ, Taylor AK, Gane LW, Godfrey TE, Hagerman PJ. Elevated levels of FMR1 mRNA in carrier males: a new mechanism of involvement in the fragile-X syndrome. American journal of human genetics. 2000;66(1):6-15. doi: 10.1086/302720. PubMed PMID: 10631132; PubMed Central PMCID: PMC1288349.

5. Gao F, Bian F, Ma X, Kalinichenko VV, Das SK. Control of regional decidualization in implantation: Role of FoxM1 downstream of Hoxa10 and cyclin D3. Scientific reports. 2015;5:13863. doi: 10.1038/srep13863. PubMed PMID: 26350477; PubMed Central PMCID: PMC4563553.

6. Stielow B, Kruger I, Diezko R, Finkernagel F, Gillemans N, Kong-a-San J, et al. Epigenetic silencing of spermatocyte-specific and neuronal genes by SUMO modification of the transcription factor Sp3. PLoS genetics. 2010;6(11):e1001203. doi: 10.1371/journal.pgen.1001203. PubMed PMID: 21085687; PubMed Central PMCID: PMC2978682.

7. Martinerie L, Manterola M, Chung SS, Panigrahi SK, Weisbach M, Vasileva A, et al. Mammalian E-type cyclins control chromosome pairing, telomere stability and CDK2 localization in male meiosis. PLoS genetics. 2014;10(2):e1004165. doi: 10.1371/journal.pgen.1004165. PubMed PMID: 24586195; PubMed Central PMCID: PMC3937215.
