# Supplementary material for: BRDT is an essential epigenetic regulator for proper chromatin organization, silencing of sex chromosomes and crossover formation in male meiosis
Source: PLoS Genet. 2018 Mar 7;14(3):e1007209. doi: 10.1371/journal.pgen.1007209 (PMC5841650; doi:10.1371/journal.pgen.1007209)
Supplement: S2 Table — (PDF) [file pgen.1007209.s009.pdf]

**S2 table**

**Primer sets used for Tiled qPCR analysis of the sub-region of the PAR hotspot**

| Number | Primer Forward | Primer Reverse |
|---|---|---|
| ***1*** | TCGCTCCTTCAGCTCTGC | AACGTTGCTTGCTTGCTAAAA |
| ***2*** | TGAGCATAAATTTCTTTTGATTCCA | AGGAAGCAATGTCTGAGTTCCA |
| ***3*** | CCCTGGAACTCAGACATTGC | GTGCAAAAGCAATCTTCATCG |
| ***4*** | AGAGGAACACTTTCTCCTGGAAG | ACCATTCATTGAGGGACTCATTT |
| ***5*** | TGCTTTTGCACAAAAATATACTTCC | GGGAATATGAGGGATGCTAAAGA |
| ***6*** | GGTAAACTACATTGCCATTGTTAGGA | GGGAATTTTATTGCTGAATGTGC |
| ***7*** | AAACTTTAAATATGCACATTCAGCAA | ACTGGACTCAGAATGATCTGGAA |
| ***8*** | TTGGTGATTGATGGTGATGC | GTGGTGTCTGTTCACGGATG |
| ***9*** | GGCTTAGGTGAACTCCCAGTG | CCCTCAACCAAATTGTGAGC |
| ***10*** | ACCCCATCCGTGAACAGAC | AATCATTTCATCTTTTCGTGATTGT |
| ***11*** | GGGAGAGAGAAACAATCACGAA | TGGCTTCTTACCCAGCTCAC |
| ***12*** | AGCTGGGTAAGAAGCCACTTG | TGCCTATACCATGCCTTTGG |
| ***13*** | GCCACTTGAGATGGCTAGGTC | CTGCCAAGGTCCCCATTT |
| ***14*** | AAGCGTCCAAAGGCATGG | TTTTCATGTAGGTGACTGCCAAG |
| ***15*** | TGAAAATAGTTCATTTCCCAGATACA | CCCCTTAGATTTCATCCTTGCT |
| ***16*** | TGCTGGATAACAGCTCTTACTGAAA | GCACTGTTTTCAGTGTTCATCCA |
| ***17*** | AAAGCGCCAGCAAGGATG | GAGTTGCCCGTTTGTTTGC |
| ***18*** | TGCTGCAAGTCCTGTTTGAC | AATGAAGGGATACATGGCAGTC |
| ***19*** | ACGGGCAACTCTGAACACA | GCAGGTAAAAACGGGAGTGG |
| ***20*** | GCCATGTATCCCTTCATTTCC | CCTGAGAAAGGCAGCCTCA |
| ***21*** | TCCATGAGCAGAGCACCA | GCACCTGCTCACCCCTTT |
| ***22*** | CACCATTGCTCAATCCCTTC | TTCTGGCTCTGGGGAGGA |
| ***23*** | CAGAAAGGGGTGAGCAGGT | CACCCAGCTGAAGCAGCA |
| ***24*** | CCCCTTCCTGGCTACTGC | TCAAGGAGAGGCTTGGAGGT |

| | | |
|---|---|---|
| ***25*** | TGCTTCAGCTGGGTGTGA | TGTCAGGCAAATTGGAGAGG |
| ***26*** | CCTCCAAGCCTCTCCTTGA | AGCACTGGGGTGAATGTCC |
| ***27*** | GGACATTCACCCCAGTGCT | GGCTCTTGAGGGGCAGGT |
| ***28*** | CCCTACCTGCCCCTCAAG | GGCACAAGTTACTGGGTGTTTG |
| ***29*** | TCAAGAGCCTTGTTCCATCC | CCGAGAGGTAATATATAGGCACAAGT |
| ***30*** | GCAGGACACAAACACCCAGT | GGAATTGGCTGAGTTCCAGA |
| ***31*** | CTCCTGAGAAATGAGGGCTTC | GCTGAAATAACAAAAGTTAAGATGGAA |
| ***32*** | TCTGGAACTCAGCCAATTCCT | GTCTTTGTGCTCAATTCATAGCTG |
| ***33*** | CAGCTATGAATTGAGCACAAAGAC | GAGGCCAGCTACAGCCTTTTA |
| ***34*** | TCATTTCTTAATAAAAGGCTGTAGCTG | AGGTAAGCTGTAGTCCTTGCTGCT |
| ***35*** | GCTGGCCTCCACATAGCC | CCAGCTTCAGGTAGAAATGTGTC |
| ***36*** | GCAGCAAGGACTACAGCTTACC | TTTCCTCACCGTTCCAAGC |
| ***37*** | AGCTTGGAACGGTGAGGAA | TGATTTTATATTTCCTAATTGGCTGTG |
| ***38*** | TTGAGCGTCAATAAGCTGAAACT | GCTTTGGGCCTCTTGCATA |
| ***39*** | TTTCAACTGTTCTGCATGTTTGA | CCAGTTCAAGCTTTCTGCACTT |
| ***40*** | CCAGATCTCTTTTCCCAAGACC | GCGTGCAGCTGTACTCCAT |
| ***41*** | CATCACCAAAGGGTACACAGG | AAAGCCCATCCTCAGTGACA |
| ***42*** | GGTTGCAAACACCCAAGC | ACATCCAGGCAGGCATTG |
| ***43*** | AGTTAACCTCCACAAGTAAGGAAGTG | TGGAACTGGCCTCAAACTGT |
| ***44*** | CCAGGCAGGGCTAAATAGTGA | CCAGCCAAGTGTCCCTAGGTAAT |
| ***45*** | TGAGTTTCCGCATCACAGC | AGCTGAATGATGTTCTTAAACAAAACT |
| ***46*** | AAGAGTGCCAACTGCTCTTCC | TCCAGAAGAGGGGATCAGATTT |
| ***47*** | GAGAGACCATATTTTCCACATAACACT | ATGAGCACACTAACAAATTGCTCTC |
| ***48*** | TTTTCAGCTTACACCGCTATCC | AAGGAGATCAGTTGTTGCTTGC |
| ***49*** | GCACACCTTTAATCCCAGCA | CTGTGTAGCCCTTGCTGTCC |
| ***50*** | ACTCCCACAACTGTAGCTTCCTT | GGAGAAAACGGACTCCCATTA |
| ***51*** | TGGAAATGGAACTCACATGGT | TGTTGGAATGTTCCCATCTCA |
